# A review of deep learning methods for ligand based drug virtual screening

**DOI:** 10.1016/j.fmre.2024.02.011

**Published:** 2024-03-11

**Authors:** Hongjie Wu, Junkai Liu, Runhua Zhang, Yaoyao Lu, Guozeng Cui, Zhiming Cui, Yijie Ding

**Affiliations:** a School of Electronic and Information Engineering, Suzhou University of Science and Technology, Suzhou 215009, China; b Yangtze Delta Region Institute (Quzhou), University of Electronic Science and Technology of China, Quzhou 324000, China

**Keywords:** Virtual screening, Deep learning, Drug discovery, Drug-target interaction, Drug-target affinity

## Abstract

Drug discovery is costly and time consuming, and modern drug discovery endeavors are progressively reliant on computational methodologies, aiming to mitigate temporal and financial expenditures associated with the process. In particular, the time required for vaccine and drug discovery is prolonged during emergency situations such as the coronavirus 2019 pandemic. Recently, the performance of deep learning methods in drug virtual screening has been particularly prominent. It has become a concern for researchers how to summarize the existing deep learning in drug virtual screening, select different models for different drug screening problems, exploit the advantages of deep learning models, and further improve the capability of deep learning in drug virtual screening. This review first introduces the basic concepts of drug virtual screening, common datasets, and data representation methods. Then, large numbers of common deep learning methods for drug virtual screening are compared and analyzed. In addition, a dataset of different sizes is constructed independently to evaluate the performance of each deep learning model for the difficult problem of large-scale ligand virtual screening. Finally, the existing challenges and future directions in the field of virtual screening are presented.

## 1 Introduction

Drug discovery, aimed at developing new drug candidates, remains a key issue in biomedicine [1]. According to statistics, it takes an average of 10–15 years and $2 billion to invest in a new drug from screening to market [2,3]. Although high throughput screening is powerful and effective, it is time and cost intensive, requires significant compound and protein data, and is dependent on testing methods for biological activity. In the past three decades, computational methods have been widely used in all aspects of the drug development process in order to optimize this process. Virtual screening is a computational approach to screen potential drug candidates from a large database of billions of ligands, greatly reducing the number of ligands screened in wet experiments and thus accelerating drug discovery [4].

In recent years, deep learning has shown excellent performance in areas such as computer vision and natural language processing, and has also been applied to computer-aided drug design [5]. Deep learning models can process large-scale complex biochemical data, and learn and exploit the implicit patterns. Deep neural networks can process the properties of atoms and bonds of compounds well while retaining structural and topology information to capture their implied features. Compared with traditional machine learning algorithms, deep learning-based virtual screening methods are able to capture more complex drug and target knowledge representations while discovering the hidden association information. However, different deep learning models have shown different performance in virtual screening. In addition, deep learning methods have encountered bottlenecks when facing the problem of ligand screening for large proteins, and how to effectively solve this problem has become a hot research topic in virtual screening.

In order to provide researchers with a better understanding of the latest advances in virtual screening and promote the application of deep learning in virtual screening, this paper provides a comprehensive analysis and description of the latest technology and research progress in this field. This review first introduces the basic concepts of virtual screening, commonly-used databases, and data representation methods. Then, various deep learning-based computational methods for state-of-the-art drug-target interaction and binding affinity prediction are analyzed and compared, including the categorization of different methods and discussion of the results. To further address the difficulty of virtual screening of large proteins, a quantitative comparison of several representative methods is extensively constructed independently with datasets of different sizes. In closing, the major issues and future directions are discussed, including novel computational strategies and ideas for employing our knowledge on virtual screening. Despite the numerous existing and emerging reviews in this area [6], [7], [8], [9], [10], [11], [12], the main contributions of this work are summarized as follows:

1) We elaborate the multiple data representation methods of both compounds and proteins, including their merits and demerits. Furthermore, our review is the first to discuss in detail substantial pre-trained model embeddings employed in this domain, which can be a novel and extra data representation and has some development potential.

2) Extensive description and explanation are provided for a vast number of deep learning-based computational models for drug virtual screening. We also compare and analyze the performance of state-of-the-art methods on several benchmark datasets.

3) We conduct extra experiments on novel datasets, which is divided from four benchmarks according to the length of proteins. To the best of our knowledge, our work is the first to explore the large-scale protein processing and encoding in virtual screening.

## 2 Overview of virtual screening

### 2.1 Problem definition

**Fig. 1:**
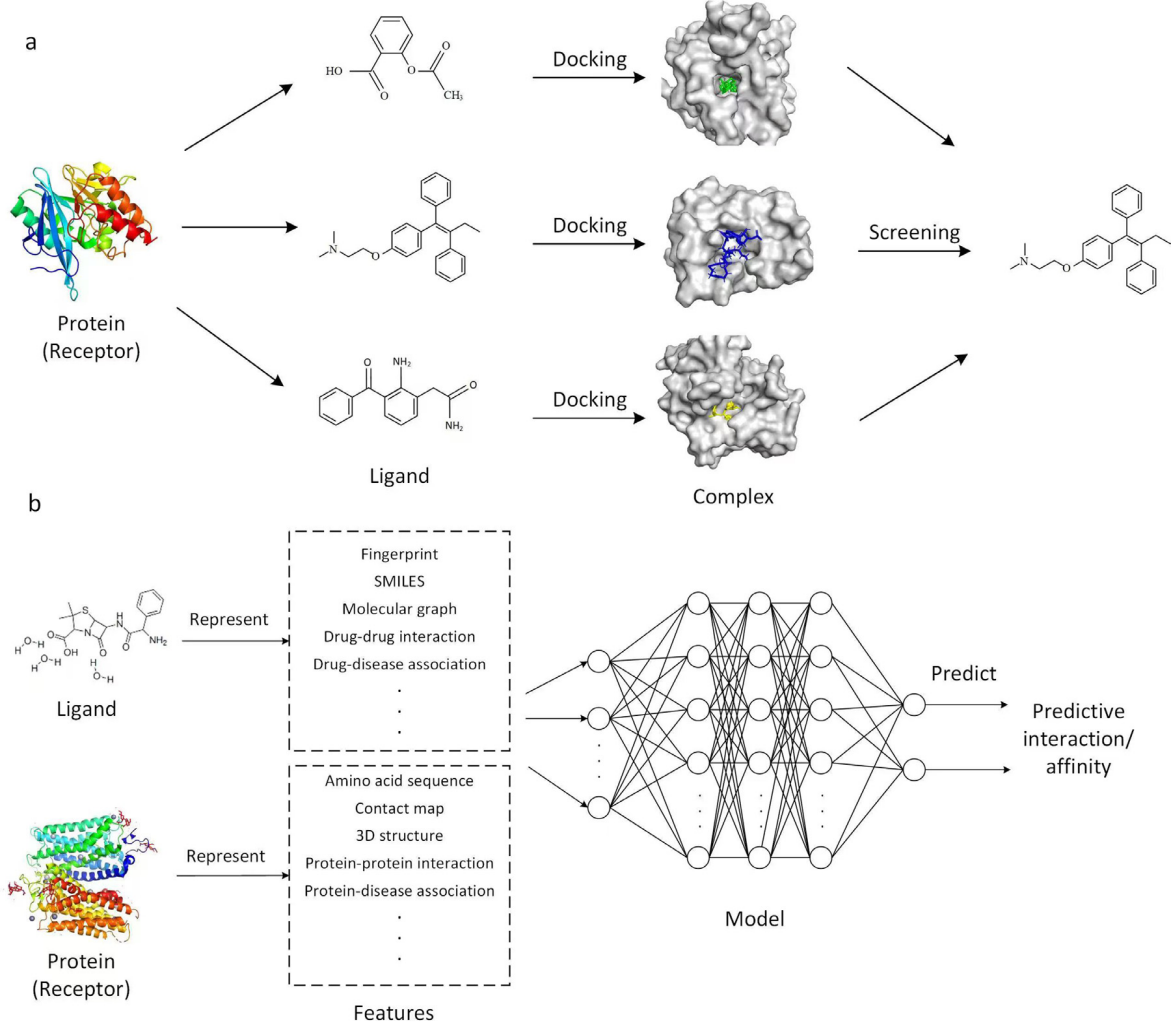
**Schematic diagram of common virtual screening methods.** (a) Receptor-based virtual screening. (b) Ligand-based virtual screening.

Virtual drug screening, or virtual screening for short, refers to the screening of candidate drug ligands for target receptor proteins from a large-scale compound ligand database by a computational model to predict possible potential drugs. The virtual screening problem, when translated into a computer problem, can be described as follows: input a ligand and receptor pair, and calculate the output of their affinity magnitudes to reduce the number of compounds actually screened and improve the efficiency of drug discovery. It should be noted that in this paper, the ligand refers to a compound molecule that has the potential to become a drug, while the receptor refers to a protein. Virtual screening methods can generally be divided into two categories: receptor-based virtual screening and ligand-based virtual screening methods, as shown in Fig. 1 below.

**Receptor-based virtual screening.** This class of methods, also known as structure-based virtual screening, starts from the three-dimensional (3D) structure of the protein, utilizes techniques such as molecular docking to investigate the characteristic properties of the binding site of the target protein and its interaction pattern with small-molecule drugs, and evaluates the binding ability of the protein and drugs according to the affinity scoring function (SF) related to the binding energy. The drugs with high predicted scores are finally selected from numerous compound molecules for subsequent bioactivity testing [13], [14].

**Ligand-based virtual screening.** Such methods usually start from ligands, analyzing the structure and activity data of known inhibitor molecules, and synthesizing the key characteristics crucial for binding efficacy, encompassing chemical, geometric, and physical properties. Subsequently, this acquired knowledge is applied to screen the ligand database, facilitating the identification of drug molecules that fulfill the specified criteria [15].

Deep learning-based virtual screening approaches usually focus on predicting the interaction and binding affinity between drugs and targets by first representing drug and target protein data as vectors or graphs using various coding methods. Then the data are processed, followed by extracting their features using deep learning models, and finally predicting the interaction or affinity between them. Predicting the binding affinity between ligand compounds and receptor proteins is a key step in ligand-based virtual screening. The prediction of drug-target interaction (DTI) and drug-target affinity (DTA) by computational methods is the focus of research on virtual screening methods based on machine learning and deep learning [16]. For virtual screening, it is a computational biology approach designed to computationally analyze a large number of compounds in order to predict their ability to bind to a specific biological target. It is a method of preliminary drug screening performed on a computer that can help researchers conduct more targeted compound testing in the laboratory. In terms of Drug-Target Interaction (DTI) prediction, it is a more narrowly defined computational biology approach that mainly consists of the prediction of interactions between drugs and multiple potential targets in an organism. It mainly uses methods such as machine learning and data mining to predict new potential interactions based on a large amount of experimental data and known information on drug-target interactions, providing a more comprehensive perspective on drug discovery. Thus, DTI prediction focuses mainly on predicting the binding of compounds to specific targets by computational methods, whereas virtual screening is broader and includes a comprehensive analysis of the interactions of multiple potential targets. DTI prediction can be viewed as a part of virtual screening as a link in the drug discovery process.

There are extensive deep learning-based methods for DTI and DTA prediction calculation. These methods are mainly divided into two different frameworks, classification task and regression task. Classification methods treat DTI prediction as a binary classification problem, where the output is whether to bind or not. Nevertheless, the simple binary classification ignores the binding affinity value which is a continuous value and important information on drug-target interactions. Binding affinity provides information on the strength of the drug-target pair interaction, which is usually expressed in terms of K_d_, K_i_ and IC_50_. Recently, there have been numerous computational approaches based on regression task background to represent drug-target protein interactions, and this method also achieves excellent performance.

### 2.2 Traditional machine learning methods

Various machine learning techniques, including regression models and classification methods such as multiple linear regression, nearest neighbor, plain Bayesian classification, support vector machines, neural networks, and decision trees have been successfully applied in virtual screening [17]. The use of multiple sets of decision trees can form a random forest, which is a widely-used integrated learning algorithm, and achieves competitive DTI prediction performance [18], [19]. However, an important issue in using decision trees for DTI prediction is the generation of features, as using nodes or edges in the structure of a drug or protein as features can lead to the loss of contextual information [20]. Moreover, the generalization ability of DTI prediction based on decision tree methods is quite limited. In addition to random forests, there are other DTI prediction methods based on tree structures. Li et al. used Bayesian methods as prior knowledge to build a model based on Bayesian additive regression trees (BART) [21]. Smhm et al. applied XGBoost to train the reduced features which showed that the XGBoost classifier outperformed the other three learning methods [22]. He et al. proposed a SimBoost model based on gradient boosting tree (GBT) to represent DTI by feature engineering to predict the combined affinity score [23]. The kernel-based approach can effectively determine the nonlinear decision boundaries of DTI and therefore is also employed in this field. Cichonska et al. introduced the KronRLS algorithm, utilizing only the two-dimensional (2D) drug compound similarity-based and the Smith-Waterman similarity representation of the protein, and calculated pairwise kernels from the drug and protein Kronecker product [24], [25]. Shi et al. proposed LASSO-DNN in which multiple LASSO models are used to integrate different combinations of protein and compound features and reduce the influence of less important features [26]. Furthermore, support vector machine (SVM)-based approaches have also been widely used for DTI prediction [27], [28], [29]. Although the kernel method is a powerful classification method, the selection of features is important for constructing decision bounds and interpretation, which is one of the major factors limiting its application.

### 2.3 Task challenges

Deep learning methods are receiving increasing attention in bioinformatics. However, the complexity of proteins, drugs and their interactions make deep learning-based virtual screening models challenging and complex, mainly for the following reasons.

**Data complexity.** Traditional machine learning frameworks use feature engineering to select relevant features for downstream tasks. In contrast, one of the major characteristics of deep learning methods is to avoid complex feature engineering and automatically learn the abstract representation of the data [30]. With the recent rise in large-scale data and increased computational power, deep learning techniques have achieved unprecedented breakthroughs in many fields, including image processing, natural language processing, and bioinformatics. The data involved in virtual screening includes not only compounds and proteins, but also complex physical, chemical and biological processes. The combination of compounds and proteins is the result of a high concentration of various factors. Therefore, drug molecules and proteins are far more complex than images, languages and other data types.

**Data annotation.** Deep learning methods require large databases for model training. Biomedical data may lack completeness and accuracy due to their noise, so there is a lack of high-quality and large-scale data with annotation. Manual annotation is costly and slow enough to fill the gap between well-labeled and unlabeled biological and chemical data. Deep learning methods for virtual screening are mainly modeled as supervised classification or regression problems, which can also be regarded as DTI and DTA prediction problems. Supervised deep learning has high demands on the quantity and quality of labeled datasets. To address the problem of insufficient amount of labeled data, researchers have started to apply unsupervised learning, semi-supervised learning or self-supervised learning to predict DTI and DTA. Additionally, unsupervised pre-training models for large text corpora have shown remarkable performance in various natural language processing tasks. Hence, some unsupervised pre-training models for learning protein amino acid sequences and drug Simplified Molecular Input Line Entry System (SMILES) representations have been proposed in recent years [31].

**Feature representation.** In addition to the lack of labeled data, existing traditional machine learning and deep learning methods also have difficulty in fully utilizing the features of ligands and proteins. Data-related features can be expressed in different forms. Drug-related features include molecular structure, functional groups, and molecular properties, etc. [32]. Protein-related features contain primary structure, secondary structure, tertiary structure, functional annotation, motifs, and various physicochemical properties [33]. Which types of features are associated with the binding mechanism between drugs and proteins, and how to select, represent, and merge features into data-driven deep learning models are significant questions that need to be investigated. In addition, large models or pre-trained models have essential prospects in drug virtual screening, where the approach for applying them effectively and rationally to the field is also a major challenge [34].

**Model selection.** Early deep learning-based virtual screening methods mainly used various types of deep neural network models, and many studies have demonstrated that they can be better applied in this field. Simultaneously, extensive recent graph and transformer-based models have also achieved significant performance improvements in virtual screening [35]. However, there is a lack of other applications of deep learning models, such as various unsupervised learning methods and generative models that lack attention and research [36].

## 3 Databases for virtual screening

In the past few years, databases in the biomedical field have gained significant improvements in both quality and quantity, which further facilitate the application of deep learning in virtual screening. This section describes a vast number of popular used databases associated with virtual screening and the benchmark datasets extracted from those databases, as shown in Fig. 1a. Specifically, based on these databases, we classify them into three categories, drug-centered databases, protein-centered databases, and integrated databases. In terms of the benchmark datasets, we divide them according to their scope of application, i.e., DTI and DTA task.

### 3.1 Drug-centered databases

In this section, we elaborate five popular drug-centered databases, which are PubChem [37], ChEMBL [38], DrugBank [39], SuperDRUG2 [40] and DrugCentral [41].

**PubChem.** PubChem is a repository for information on chemical substances and their related biological activities [37]. PubChem is the world's largest free database of chemical information, including chemical structures, physicochemical properties, biological activities, patents, health, safety, and toxicity data. PubChem contains 2D and 3D structural information on compounds, as well as information on interacting proteins from various biochemical experiments and literature. The database is composed of three subsets: Substance, Compound and BioAssay. Substance serves as the primary repository for chemical information provided by individual data contributors. The Compound database contains the unique chemical structures extracted from the Substance database. The BioAssay database, meanwhile, stores all biological-related data accompanying these chemical substance data.

**ChEMBL.** ChEMBL is a large and open-access drug discovery database designed to collect medicinal chemistry data and knowledge from the drug research and development process [38]. ChEMBL contains information on compounds and their bioassay results extracted from medicinal chemistry journals, approved drugs, and clinical development reports. Established by the European Molecular Biology Laboratory (EMBL) - European Bioinformatics Institute in 2002, ChEMBL has undergone its latest update in 2018. Currently, it contains over 1.9 million chemical compounds, including more than 10,000 drugs and over 12,000 targets recorded in ChEMBL.

**DrugBank.** DrugBank is one of the most widely used databases and has become a reliable resource for drug reference information [39]. Initially released in 2006, DrugBank serves as a bioinformatics and cheminformatics database which provides comprehensive drug data and protein information. The DTI relationships in DrugBank are primarily extracted from textbooks, published articles, and other chemical databases.

**SuperDRUG2.** SuperDRUG2 is established as a data repository which offers all crucial features of approved and marketed drugs [40]. The drug items in SuperDRUG2 are classified into two categories: small molecules and biological/other drugs. Several public resources, including US FDA, CFDA, EMA, and others, were used for drug collections. In addition to this mentioned information, SuperDRUG2 also provides 2D and 3D structure information of small molecule drugs, drug side effects, drug-drug interactions, and drug pharmacokinetic parameters.

**DrugCentral.** DrugCentral is a comprehensive database that focuses on drug compilation [41]. Released in 2016, this database contains FDA-approved active pharmaceutical ingredients i.e., drugs and other regulatory agency-approved drugs. For each drug, this database incorporated structure information, bioactivity and regulatory records, as well as pharmacologic actions and indications. The drugs in DrugCentral are classified into three categories: small molecule active ingredients, biological active ingredients and others.

### 3.2 Protein-centered databases

Five protein-centered databases are introduced in this category, including PDB [42], UniProt [43], BRENDA [44], PDID [45] and Pharos [46].

**PDB.** Protein Data Bank (PDB) is the primary source of 3D structural information on proteins and protein-ligand complexes [42]. As the first large-scale open-access database in biology and medicine, PDB provides 3D structures of macromolecules determined experimentally, including numerous proteins, nucleic acids, and a subset of ligand-protein complexes and nucleic-acid-protein complexes. Most of the structure in the PDB was determined by X-ray diffraction, but approximately 10% of the structure was determined by protein NMR. When X-ray diffraction is used, an approximation of the coordinates of the protein atoms is obtained, while when NMR is used, the distances between pairs of protein atoms are estimated. The final conformation of the protein is obtained from NMR by solving the distance geometry problem.

**UniProt.** UniProt is another representative protein database, whose data are mainly derived from protein sequences obtained from genome sequencing projects [43]. UniProt is currently the international protein sequence database with more complete sequence data and richer annotation information. It contains a large amount of information on the biological functions of proteins from the literature. At present, UniProt has included 560,000 protein data in Swiss-Prot.

**BRENDA.** BRENDA is a comprehensive enzyme database which was first constructed in 1987 [44]. It contains about 84,000 enzymes and their corresponding enzyme-ligand information, manually evaluated and extracted from nearly 140,000 literature references. In BRENDA, all chemical compounds related to enzyme catalyzed reactions are designated as 'ligands', including substrates, products, activators, inhibitors, and cofactors. Overall, approximately 205,000 enzyme ligands were collected and stored in the associated ligand database. Additionally, BRENDA provides download functionality for users to obtain all information data.

**PDID.** PDID, released in 2014, comprehensively annotates all known and predicted drug-protein interactions for the entire structural human proteome [45]. The known interactions were extracted from DrugBank [39], BindingDB [50] and PDB [42].

**Pharos.** Pharos is a platform created to present the data collated in the Target Central Resource Database (TCRD) [46]. TCRD, initially proposed for the identification of novel druggable proteins, constitutes a comprehensive repository of information. The data hosted in TCRD originate from diverse sources, including biomedical literature, gene expression data, associations between diseases and phenotypes, bioactivity data and DTI data.

### 3.3 Integrated databases

Integrated databases curated to provide additional annotations and information have also been developed. In this paper, we enumerate six databases that fall into this group. Among these databases, some may not have been explicitly designed as DTI or DTA databases, but the information they house can still be exploited for virtual screening research. More specifically, databases belonging to this category include KEGG [47], STITCH [48], SuperTarget [49], BindingDB [50], PDBBind [51] and BindingMOAD [52].

**KEGG.** KEGG is a comprehensive database which provides multiple types of information centered on genes and genomes [47]. The entire database can be summarized into four major categories. The first category is systems information, which includes three databases. The second category focuses on genomic information. The third category, which is mainly about chemical information, features five databases. The final category is health information, which includes four databases. Among them, the KEGG DGROUP database contains information regarding drug interaction networks, including DTIs, drug metabolism, and indirect interactions with enzymes and target genes.

**STITCH.** STITCH is a repository that archives information on protein-compound interactions [48]. The interaction data are procured from computational predictions, other databases including PubChem, and literature citations. The initial release of STITCH occurred in 2008.

**SuperTarget.** SuperTarget is a repository that constitutes information on DTI, drug metabolism, pathways, and Gene Ontology (GO) terms [49]. It further includes medical indications and adverse drug effects. The knowledge of DTI within SuperTarget was extracted through text mining from 15 million public literatures listed in PubMed. Moreover, potential DTIs were extracted from Medline. Additionally, relationships of DTIs from other databases, including DrugBank, KEGG, and PDB, were used to acquire and supplement any missed DTIs.

**BindingDB.** BindingDB is another publicly accessible database that mainly collects the interaction affinities between drug target proteins and drug-like small molecules [50]. All of these data were extracted from scientific literature and patents. Furthermore, other databases, including ChEMBL and PubChem, are also linked with BindingDB.

**PDBBind.** The PDBbind database combines all the biomolecular complexes in the PDB database and experimentally derived binding affinity data [51]. PDBbind was first released in 2004 with the goal of bridging the gap between protein structural information and energetic properties. The data stored in PDBbind were classified based on the biomolecular complex data from PDB. Subsequently, binding affinity data were collected from the associated literature accompanying each PDB entry.

**BindingMOAD.** BindingMOAD (Mother of All Databases) is another repository which focuses on providing combined high-quality structural and binding affinity data [52]. The latest BindingMOAD (release 2019) archives 38,702 well-resolved protein-ligand crystal structures, with ligand annotations and protein classifications, of which 15,964 are associated to experimental affinity data with biologically-relevant ligands (Table 1).

**Table 1:** **Comparison of statistical information of databases**.

Type	Databases	Drugs	Proteins	Interactions	Latest Updates	Availability
Drug-centered	PubChem	115,348,331	186,035	N/A	Sep 2023	https://pubchem.ncbi.nlm.nih.gov/
	ChEMBL	2,399,743	15,398	N/A	May 2023	https://www.ebi.ac.uk/chembl/
	DrugBank	16,558	5,298	18,984	Jan 2023	https://go.drugbank.com/
	SuperDRUG2	4,605	4,456	N/A	Mar 2018	http://cheminfo.charite.de/superdrug2
	DrugCentral	4,959	N/A	N/A	Sep 2022	https://drugcentral.org/
Protein-centered	PDB	N/A	210,836	N/A	Oct 2023	https://www1.rcsb.org/
	UniProt	N/A	570,157	N/A	Apr 2023	https://www.uniprot.org/
	BRENDA	> 20,500	>84,000	N/A	Feb 2023	https://www.brenda-enzymes.org/
	PDID	51	3,746	16,800	Apr 2015	http://biomine.cs.vcu.edu/PDID/
	Pharos	355,932	20,412	N/A	Apr 2023	https://pharos.nih.gov/
Integrated	KEGG	19,136	N/A	N/A	Oct 2023	https://www.kegg.jp/
	STITCH	430,000	9,643,763	1.6 billion	Jan 2016	http://stitch.embl.de/
	SuperTarget	196,000	>6,000	> 330,000	Mar 2023	http://bioinformatics.charite.de/supertarget
	BindingDB	1,194,492	9,180	2,791,311	Sep 2023	https://www.bindingdb.org/
	PDBBind	11,762	3,782	17,679	Jan 2020	http://www.pdbbind.org.cn/
	BindingMOAD	20,387	11,058	41,409	Jun 2019	https://bindingmoad.org/

### 3.4 DTI task datasets

The drug and protein database allows researchers to quickly search for information on the currently reported ligand compounds and their activities of a given target protein, which can save time during literature review and compound data collection, and further facilitate the development of drug discovery and design. For machine learning tasks, including deep learning-based virtual screening, evaluation of models and algorithms on benchmark datasets is essential. Based on the databases presented above, researchers have constructed numerous benchmark datasets for DTI and DTA prediction. Table 2 below illustrates the basic information on benchmark datasets, including the type of dataset, number of ligand compounds, proteins, and interactions included; data source, deep learning method using the dataset, and their availability through websites.

**Table 2:** **Comparison of statistical information of benchmark datasets.** The table depicts some widely used databases with the type of dataset, the number of drugs, proteins, and interactions, the data source, the deep learning methods using the dataset, and their availability through websites.

Type	Datasets	Drugs	Proteins	Interactions	Data source	Deep learning methods using dataset	Availability
DTI	DUD-E	22,886	102	22,800	PubChem, ZINC	[56,^109^,^79^,^116^,123]	http://dude.docking.org/
	MUV	1,360	17	1,360	PubChem, ZINC	[109,^116^,123]	https://www.tu-braunschweig.de/pharmchem/forschung/baumann/muv
	Human	2,726	2,001	6,728	DrugBank, Matador	[56,^59^,^79^,^124^,^125^,^136^,137]	https://github.com/masashitsubaki/CPI_prediction/tree/master/dataset
	*C.elegans*	1,767	1,876	7,786	DrugBank, Matador	[56,^125^,^132^,^136^,137]	https://github.com/masashitsubaki/CPI_prediction/tree/master/dataset
	BindingDB	53,253	1,696	70,965	BindingDB	[55,^57^,^59^,^108^,^79^,^124^,132]	https://www.bindingdb.org/
	DrugBank	6,655	4,294	35,022	DrugBank	[56,^58^,^107^,^111^,^116^,^118^,^120^,133]	https://go.drugbank.com/
DTA	Davis	68	442	30,056	PubChem, UniProt	[78,^92^,^106^,^110^,^113^,^115^,^117^, 124,125,131,133,134]	https://github.com/hkmztrk/DeepDTA/tree/master/data
	KIBA	2,111	229	118,254	ChEMBL, UniProt	[78,^92^,^106^,^110^,^113^,^115^,^117^,^124^,^125^,^131^,^133^,134]	https://github.com/hkmztrk/DeepDTA/tree/master/data
	PDBbind	3,672	1,287	4,446	PDBbind, BindingDB	[55,^57^,^110^,112]	http://www.pdbbind.org.cn/

Classification methods consider DTI prediction as a binary classification problem, where the output is whether to bind or not. This category of methods requires both drug, protein and their interaction data.

**DUD-E.** DUD is an acronym for Directory of Useful Decoys, where decoys or negative samples are active molecules with similar physicochemical properties but different 2D structures [53]. DUD-E incorporates experimentally validated active compounds interacting with specific target proteins and meticulously selected decoys—inactive compounds sharing similar physicochemical properties with actives. This dataset is crucial for evaluating models, assessing their proficiency in distinguishing true positives (active compounds) from false positives (decoys). It is commonly used in classification tasks for DTI prediction, evaluating the model's ability to rank active and inactive compounds (decoys). However, it's important to note that some studies have demonstrated the presence of hidden analogue bias and decoy bias in this dataset.

**MUV.** MUV, which stands for Maximum Unbiased Validation, was obtained from PubChem BioAssay database [54]. The MUV dataset was constructed mainly to minimize two different biases commonly found in existing virtual screening benchmark datasets, i.e., artificial enrichment and simulation bias. Unlike the DUD-E dataset, MUV takes a distinctive approach by refraining from utilizing computational decoys as negative samples, opting instead for experimental validation. One key strength of the MUV dataset lies in its experimental validation for both positive and negative samples, utilizing bioactivity data from the PubChem database. This ensures a more accurate representation of the true bioactivity status of compounds, enhancing the reliability of the dataset. The MUV dataset contains 17 target proteins with 30 active and 15,000 inactive molecules for each target. This diversity of target proteins offers a broad representation of biological contexts, enabling researchers to evaluate model performance across a range of specificities. Moreover, the fixed number of active compounds (30) and inactive compounds (15,000) per target in the MUV dataset might not entirely encapsulate the variability observed in real-world scenarios. In practical drug discovery contexts, the distribution of active and inactive compounds can significantly differ between targets, potentially limiting the dataset's ability to fully represent the diverse landscape of molecular interactions for all target proteins.

**Human, *C.elegans.*** In addition to the aforementioned benchmark datasets, Liu et al. [55] meticulously curated the Human [55] and C. elegans [56] datasets using a systematic framework. This approach assumes that compounds similar to known ones interact with proteins sharing similarities with known target proteins. Dissimilarity rules for proteins and drugs were carefully defined and applied to identify reliable negative samples. To bolster robustness, a threshold on the number of predicted interactions between the target protein and negative candidate compounds was introduced. These datasets have demonstrated remarkable success in various DTI classification predictions.

**BindingDB.** In 2018, Gao et al. utilized the BindingDB database, which contains 1.3 million data records, to construct a binary classification dataset [57]. The dataset, consisting of 39,747 positive examples and 31,218 negative examples, was created based on specific criteria: the presence of chemical and protein identifiers, availability of chemical structure represented by SMILES, inclusion of protein sequence and Gene Ontology annotations, and the primary measure of binding effectiveness, IC_50_ values. The dataset further restricts chemical molecule weight to less than 1,000 Da, categorizing records as positive if IC_50_ is less than 100 nm and negative if IC_50_ exceeds 10,000 nm. They recorded all drug molecules with PubChem CIDs and protein sequences with UniProt IDs, classifying each record into positive or negative samples according to its IC_50_ value, providing a comprehensive binary classification dataset for further analysis.

**DrugBank.** In the DTI prediction study by Zhao et al. [58], they utilized the DrugBank database [39] to extract drug and protein data, forming the basis for constructing their experimental dataset. The dataset employed in the study corresponds to the January 2020 release of the DrugBank database. To ensure data quality, they systematically removed inorganic compounds, very small molecule compounds, and drugs for which the RDKit package could not recognize the SMILES code, resulting in a refined dataset comprising 6,655 drugs, 4,294 proteins, and 17,511 positive drug-target pairs. In line with the methodology presented by Huang et al. [59], the researchers sampled from unlabeled drug-protein pairs to generate negative samples. This strategic sampling approach aimed to create a balanced dataset with an equal distribution of positive and negative samples, contributing to the robustness and fairness of their DTI prediction analysis.

### 3.5 DTA task datasets

The regression task aims to predict the affinity values of drugs and targets, which provide information on the strength of the drug-target pair interaction, which is usually expressed in terms of K_d_, K_i_, and IC_50_ metrics. Davis [60] and KIBA [61] are the two most commonly used DTA prediction datasets.

**Davis.** The Davis dataset is a comprehensive compilation of experimentally derived measurements specifically concentrating on kinase protein families and their corresponding inhibitors [60]. Within this dataset, selectivity measurements and K_d_ values are meticulously provided for drug-protein interactions. Notably, it prominently features drug-target pairs with an affinity value of 5, totaling 20,931 entries, while the rest mainly fall within affinity values 6 and 7. This dataset stands out for its targeted exploration of kinase interactions, offering valuable insights into binding affinities and selectivity patterns of kinase inhibitors. The inclusion of dissociation constant values enhances the dataset, offering quantitative insights into interaction strength, emphasizing a deliberate focus on moderate binding strength for physiological relevance in drug-protein interactions.

**KIBA.** On the other hand, the KIBA dataset was derived from the KIBA scoring method, which combines kinase inhibitor bioactivities from different sources, including K_d_, K_i_, and IC_50_ [61]. The KIBA scoring method scores the affinity values of the drugs and proteins by using the statistical information contained in the K_d_, K_i_, and IC_50_ to optimize the consistency between them. The KIBA dataset initially The KIBA dataset initially consisted of 467 targets and 52,498 drugs, which were filtered to produce a total of 229 proteins and 2,111 drugs even though it only contained drugs and targets with at least 10 interactions. The affinity values in the KIBA dataset were mainly distributed between 10 and 13, with most of them being around 11. In addition, Öztürk et al. [62] suggested that for 99% of the protein pairs, the Smith-Waterman (S-W) similarity between proteins in the KIBA dataset is at most 60%. In contrast, in the Davis dataset, 92% of the protein pairs have no more than 60% target similarity. The above statistics show that both datasets are non-redundant. Due to their excellent quality, Davis and KIBA have become widely used DTA datasets.

**PDBBind.** In the current release, PDBbind (v. 2019) provides binding data for a total of 23,496 biomolecular complexes, including 19,443 protein-ligand complexes [51]. In a study by Gao et al. [57], they merged the BindingDB dataset with the refined PDBbind dataset, resulting in 4,446 pairs with both affinity labels (K_i_ or K_d_) and atomic-level contacts. Encompassing interactions between 1,287 proteins and 3,672 compounds, the dataset spans diverse molecular interactions across various protein classes, including nuclear receptors, G protein-coupled receptors, ion channels, and enzymes categorized into distinct EC classes. This curated dataset also incorporates affinity-labeled compound-protein pairs derived from BindingDB, further enhancing its comprehensiveness. Compound and protein data are formatted as canonical SMILES and FASTA sequences, respectively, with additional preprocessing for compound graph representation. The dataset's interpretability is bolstered by high-resolution atomic contacts obtained from compound-protein cocrystal structures in PDB, providing valuable insights into the molecular interactions underpinning drug-protein binding.

## 4 Data representations

Virtual screening requires data on ligand drugs, target proteins, and drug-target interactions. Representation of these data in a computer-readable form is a major step in virtual screening and a major challenge for bioinformatics [63], [64]. Fig. 1b illustrates the drug and protein representations.

### 4.1 Drug data representation

Effective ligand representation and accurate property prediction are the key tasks in drug discovery work. The molecular formula is a common representation of ligand molecules. However, due to its lack of structural information, this representation makes it difficult for deep learning models to capture its features and predict its properties [65]. Currently, there are more advanced representations of ligand molecules. The main methods for representing ligand compounds in a computer-readable form are strings, fingerprints and graphs.

**Strings.** The most widely used string descriptor is the SMILES [66]. This encoding method uses characters to describe the structure of a compound molecule, and accesses nodes and edges by a depth-first traversal algorithm, which linearizes the molecular graph of the compound. The uniqueness of the SMILES encoding for each molecule structure depends on the normalization algorithm used to generate it and is known as the canonical SMILES. In addition, the SMILES Arbitrary Target Specification (SMARTS) is an extension of the SMILES encoding, which allows the use of wildcards to represent atoms and chemical bonds, allowing a better display of compound substructures [67]. Although the SMILES string representation is concise and fast, it still does not fully capture the spatial relationships between compound atoms. For example, in a benzene ring, each carbon atom has different relationships with other atoms and is located at different positions in the molecule, but the distinction between each of them cannot be well distinguished by SMILES coding. Moreover, they may have different properties. Therefore, the use of SMILES strings alone may result in the loss of some important structural information and is not sufficient to predict some properties of the molecule.

**Fingerprints.** Fingerprinting is another sequence-based representation of molecules, which contains some molecular structure information compared to SMILES strings. Molecular fingerprint encodes a compound as a binary vector by comparing it with a substructure. The fingerprint is represented by 0 and 1 for the absence and presence of substructures, respectively. Extended Connectivity Fingerprints (ECFP) are the most widely used molecular fingerprints for constructing QSAR models. In the cheminformatics software RDKit [68], ECFPs are also called Morgan fingerprints because the core idea of ECFP comes from Morgan's algorithm. The algorithm can assign a unique identifier to each atom and iteratively update the atomic identifier using a hash function by accumulating information from neighboring nodes.

**2D Graphs.** The structural and topology information contained in the 2D data of a drug molecule is essential for molecular property prediction and other downstream tasks. Graphs, based on the 2D structure of compounds have also been successfully applied to describe molecular data, which can reflect the chemical and biological properties of molecules effectively and distinctly [69]. In order to employ graph-based descriptor methods, it is necessary to convert molecules into graph data form. The current common approach is to represent ligand molecules as undirected graphs where nodes denote atoms and edges denote chemical bonds, using two types of matrices: feature matrix and adjacency matrix for representation. The feature matrix contains the characteristics and prior knowledge of each node in the dimension of N×D, where N is the number of nodes and D is the number of defined features; the adjacency matrix is used to describe the structure of the molecule in the dimension of N×N. Moreover, some molecular graph construction approaches introduce edge features that can better describe the global structure and the spatial relationships between atoms [70,71]. Using the graph-based representation, the obtained matrix can be used as the input to graph neural networks (GNN). With the recent rapid development of various types of GNN, it is possible to collect information about neighboring nodes more directly and efficiently, and thus be able to capture the spatial relationships between atoms within a molecule.

**3D Graphs.** 3D geometries depict the spatial organization of atoms in a molecule within 3D space, where each atom is linked to its type and coordinates along with optional geometric features or attributes. The merit of using 3D geometry is that the conformational information is essential for numerous molecular properties, particularly quantum properties. Furthermore, it is also effective to directly adopt stereochemical information from the given 3D structures. Multiple methods [72], [73], [74] have been proposed to discover mechanisms and principles on 3D molecular structures, which enable the graph representations to follow specific physical symmetries. Nevertheless, to the best of our knowledge, there is still a lack of 3D drug molecule representation methods for virtual screening. The main reason for this phenomenon is the complexity of 3D data and the under-exploitation and under-utilization of the relevant 3D GNNs. In conclusion, 3D graph representations for drug molecules have potential in virtual screening and deserve further development and expansion by researchers.

### 4.2 Protein data representation

Proteins are organic macromolecules involved in various life activities and are composed of different amino acid sequences, which in turn form their unique 3D folding structures, resulting in orderly to disordered and conformational changes [75]. Understanding the sequence-structure-function relationship of proteins is a central issue in protein biology and is essential for investigating disease mechanisms, protein design and drug discovery [76]. The commonly used methods for protein data representation include one-dimensional (1D) strings, 2D, and 3D graph structures.

**Strings.** The complete sequence of a protein, often referred to as the primary structure of a protein, is the order in which amino acids are arranged in a protein. All 20 amino acids in the protein primary structure can be encoded as a single letter, so one-hot encoding is usually used to represent the protein sequence [77]. One-hot encoding converts the characters representing the amino acid sequence into a binary vector, which can represent the protein primary structure briefly and efficiently.

**2D Graphs.** Although sequence is an effective way to store information about the primary structure of a protein, it cannot provide information on the 3D structure of a protein. For this reason, the protein structure can usually be converted into a spatial graph with chemical properties, which contains atomic or residue nodes and edges [77]. Currently, the widely used protein graph representations include 2D and 3D graphs.

Jiang et al. first proposed the utilization of the 2D contact map of proteins as its representation [78]. Each protein includes hundreds of amino acid residues; however, the connection between residues is only a long chain, which does not contain any spatial information. Therefore, the contact map is introduced in this method. The contact map is a representation of the protein structure, which is a 2D representation of the 3D structure of the protein and can also be used as an output for protein structure prediction. In addition, the output contact map is usually in the form of a matrix, which is exactly the same as the adjacency matrix in GNN, and this provides an efficient way to combine two data sources together. In this method, a threshold of 0.5 is set to obtain a contact map of L×L, where L is the number of nodes (residues). By this method, the spatial and structural information of the protein can be well preserved. Inspired by this work, a large number of subsequent studies have used contact or distance maps as a protein representation, and in this way have demonstrated the validity of the 2D graph structure of proteins in virtual screening [79], [80], [81], [82], [83].

**3D Graphs.** A major issue with the PDB database, the main source of protein data, is that the number of proteins with structural features is much smaller than the number of proteins with determined amino acid sequences [83], [84], by which the use of protein structural information for drug discovery is thus limited [85]. Nevertheless, the recent impressive performance of AlphaFold [86] and AlphaFold2 [87] in protein structure prediction has demonstrated that protein evolutionary information can be used to predict protein 3D structures from protein sequences very effectively, and models that can learn protein 3D structure information are becoming more sophisticated. Considering that 2D contact or distance maps are only an approximate abstraction of protein 3D structures and cannot accurately describe the complex tertiary structural features of proteins, deep learning-based virtual screening models that partially use protein 3D map structures have recently emerged with great success. In 2022, AttentionSiteDTI [88] obtained protein 3D structure from the PDB database and computationally obtained the binding pocket of each protein, constructing a protein binding pocket map at the atomic level by using atoms in amino acids as points and chemical bonds as edges. Pandey et al. [89] constructed a protein 3D structure graph by both PDB and AlphaFold databases, and demonstrated that the predicted protein structures by AlphaFold could enhance the virtual screening performance. Furthermore, Wang et al. [90] and Shen et al. [91] constructed residue-level protein graphs, in which amino acid residues are nodes of the graph, and the distance between residues is used to determine whether they are connected into edges. In summary, compared with the traditional way of encoding amino acid sequences of proteins using strings, the protein representation based on the 3D graph structure can better capture its complex spatial structure, so as to further predict the binding sites of proteins and ligands and other information, which can improve the model prediction accuracy and generalization ability, and provide the model with superior interpretability. It can be observed that the application of protein 3D structures is a major trend in the field of virtual screening. In the future, with the development of GNNs and the improvement of computational power, the virtual screening method based on protein 3D structure has a good development space, which requires further research and improvement.

### 4.3 Pre-trained model embeddings

#### 4.3.1 Drug pre-trained models

Extracting feature representations for drug molecules is critical to applying deep learning methods to a broad scope of downstream molecular tasks. Tremendous efforts have been devoted to drug molecular pre-trained models, where deep neural networks are pre-trained on large-scale molecular databases and then fine-tuned over specific downstream tasks. In this section, we briefly list four classic drug pre-trained models which have been applied into DTI or DTA prediction tasks.

**Mol2vec.** Inspired by the Word2vec models in natural language processing (NLP), Mol2vec was developed by learning representations of molecular substructures that point in similar directions for chemically related substructures [92]. In Mol2vec, molecular substructures were extracted using the Morgan algorithm where context represents words and complete molecules are analogous to sentences. Novel compounds can be described by summing the substructure vectors retrieved from a pre-trained Mol2vec model. Due to its effectiveness and usefulness, some virtual screening methods have applied Mol2vec to obtain better information on molecular properties [83], [93].

**Smiles Transformer.** SMILES Transformer learns molecular fingerprints representations through unsupervised pre-training of the sequence-to-sequence model [94]. Similar to the autoencoder, the model architecture is a transformer-based encoder-decoder network and learns the embeddings by reconstructing molecules represented by strings.

**ChemBERTa.** ChemBERTa adopted sequence-based pre-training strategies on string-based molecular data, i.e., SMILES [95]. In sequence-based pre-training, ChemBERTa mask random characters in the SMILES strings and then recover them based on the output of the transformer of the corrupted SMILES strings. In downstream molecular property prediction task, ChemBERTa obtained competitive performance on benchmark datasets.

**TrimNet.** TrimNet is a graph-based method which employs a novel triplet message mechanism to learn molecular representations efficiently [96]. In particular, TrimNet explicitly removes the matrix mapping of edge features and adopts a triplet message mechanism to calculate messages from atom-bond-atom information. TrimNet is evaluated on various molecular property predictive tasks, including DTI task, which achieves the new state-of-the-art performance.

#### 4.3.2 Protein pre-trained models

Protein representation learning is an increasingly popular domain of deep learning and bioinformatics research. Semi-supervised learning serves as an important paradigm in protein representation learning due to the high cost of acquiring supervised protein labels, but the current literature is fragmented in terms of datasets and standardized evaluation techniques. The 1D sequence of a protein determines the 3D structure of the protein. The number of known protein sequences is much more than the number of known protein structures, which are much more numerous. Understanding the structure helps to resolve the protein function. Pre-trained language models of proteins help to mine the large amount of unannotated sequence data and aid in the subsequent study of proteins [97]. Here, four pre-trained models for protein representation learning are introduced, including Tape [98], ProtTrans [99], ESM [100], and ESM-2 [101].

**Tape.** To facilitate progress in protein modeling and representation learning, the Tasks Assessing Protein Embeddings (TAPE) [98] are introduced, a set of five biologically relevant semi-supervised learning tasks spread across different domains of protein biology. TAPE encodes each amino acid into a feature vector. For each vector, it is contextual and includes the sequence information from the input protein sequence.

**ProtTrans.** Protein language models use amino acids as tokens and entire protein sequences as sentences. First, ProtTrans self-supervise the prediction of masked tokens by known sequences, a step that uses only protein sequences without any annotation as input [99]. Then, the learned embeddings of ProtTrans are extracted and used as input to migrate to supervised residue-level or protein-level training tasks. The experiments on downstream tasks demonstrate that embeddings extracted from the last protein language model layers captured constraints relevant for protein structure and function.

**ESM.** ESM explores natural language migration of protein sequences across domains by training high-capacity Transformer language models on evolutionary data [100]. ESM investigated deep learning across evolution in the largest database of protein sequences, training a contextual language model of 86 billion amino acids from 250 million sequences. The high-capacity network learnt representation space from sequences reflecting multiple levels of biological structure, including amino acids, proteins and evolutionary homology. Information about secondary and tertiary structures is internalized and represented in the network.

**ESM-2.** ESM-2 demonstrates how protein structure can be directly inferred at the full atomic level from primary sequences [101]. Specifically, similar to previous studies, ESM-2 was trained to predict features of amino acids that were randomly masked out of the protein sequence. When the approximate parameters were raised to the tens of billions level, the fidelity of the protein sequence modelling improved considerably when it was done, and this training also enabled the language model to generate protein structures. This drives an order-of-magnitude acceleration of high-resolution structure prediction, leading to macro genomic protein.

To the best of our knowledge, multimodal learning in virtual screening mainly contains the introduction of pre-trained models and their modal interaction with other data representations. Introducing pre-trained language model features does enrich the representation of entities to some extent and in this way improves performance, but only a small amount of work has investigated the relevant aspects of multimodal learning, and this direction needs to be further extended and discovered by future researchers, especially the method of fusion and interaction of multimodal data and other modality of drug and protein entities [81], [82], [83], [89], [138].

## 5 Deep learning-based virtual screening methods

**Fig. 2:**
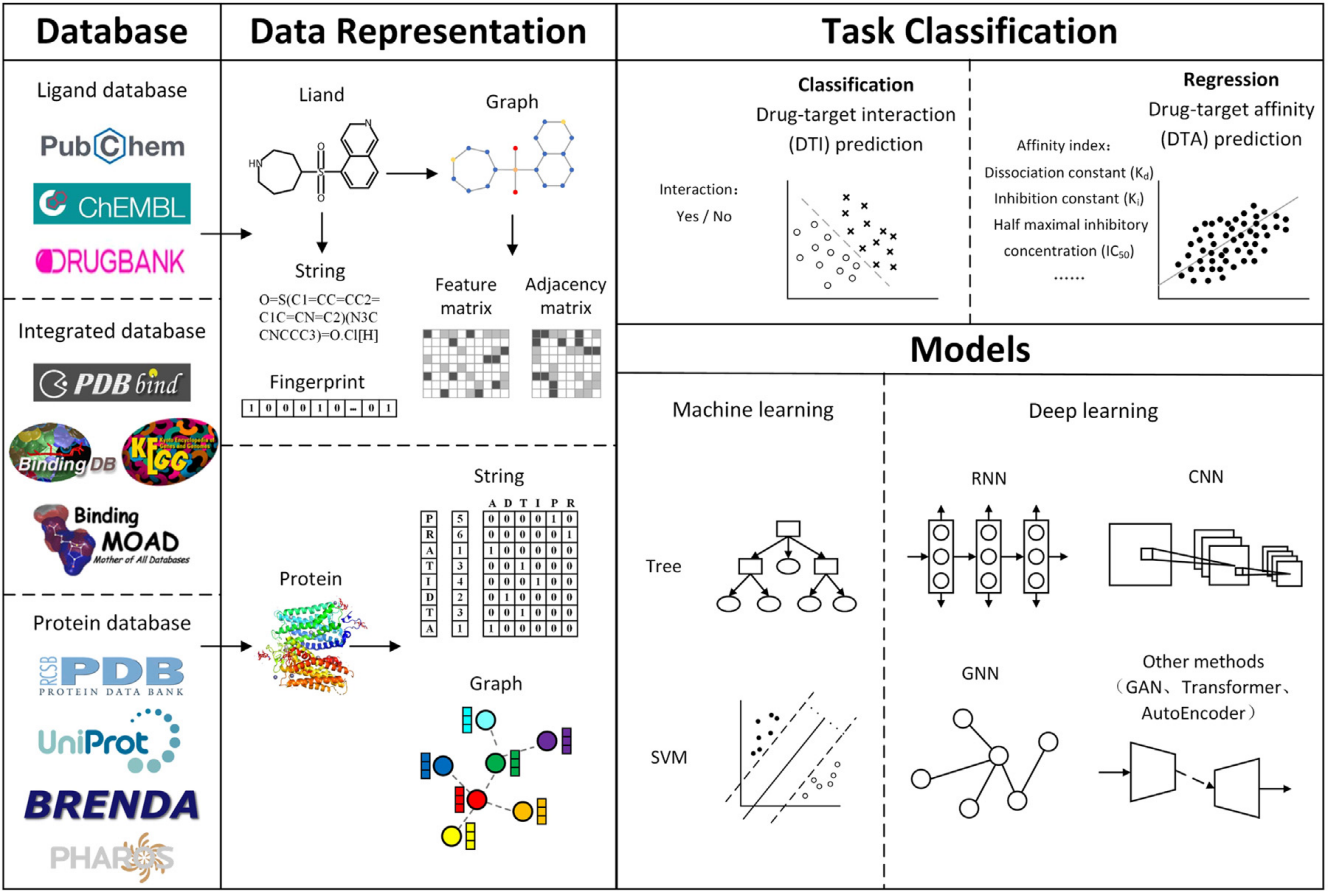
**Overview of drug-target interactions/affinity prediction**.

Recently, deep learning techniques have developed rapidly and achieved great success in virtual screening, especially in computational methods for DTI and DTA prediction. Currently, most deep learning-based models for DTI and DTA prediction use representation methods for ligand drugs and proteins, respectively, directly using their representations as features, encoding them using various types of deep learning models, extracting information from them, and combining the respective representations to predict interactions or binding affinities. In this section, we classify and study the recent DTI and DTA prediction methods from the perspective of different deep learning models, including convolutional neural network (CNN), recurrent neural network (RNN), GNN and other emerging models. It is worth noting that various representative DTI and DTA methods published in international authoritative journals and conferences in recent years have been compared and analyzed. The overall framework of the deep learning-based DTI and DTA prediction computational methods is shown in Fig. 2.

### 5.1 CNN-based methods

CNNs use a series of convolutional and pooling operations to extract relevant features from input data and were originally developed for processing and analyzing image data. The input data of CNN are usually in 2D form. When drugs and proteins are utilized as inputs, their linear representations need to be converted into 2D structures, which can usually be obtained by one-hot encodings.

Ozturk et al. first applied CNNs to DTA prediction. In their proposed DeepDTA model, CNNs were used to extract low-dimensional features of drugs and proteins, respectively [62]. The obtained feature vector representations were fed into a fully connected layer to calculate the binding affinity. Later, they added new features, including motif and domain information on proteins, and the proposed WideDTA [106] to obtained better performance compared to DeepDTA on two benchmark datasets. Lee et al. proposed DeepConv-DTI, using CNNs to convolve amino acid sequences of different lengths as an approach to obtain local residue patterns of proteins [107]. Rifaioglu et al. first used a 2D image structure to represent the drug, reducing the information loss during data conversion [109]. The research proposed by Zheng et al. used the 2D distance map of proteins as input and a CNN-based Visual Question Answering (VQA) system with a linear representation of drug molecules as query conditions to obtain the answer to whether the queried drug and protein interact with each other [79]. Wang et al. proposed DeepDTAF, which incorporates protein binding pockets as input features to integrate local and global contextual information [112]. Zhao et al. proposed HyperAttentionDTI, in addition to using CNN as drug and protein encoders, designed a new attention mechanism by assigning an attention vector to each amino acid-atom pair, enabling a more comprehensive representation of amino acid and atom interactions [58]. Summaries of CNN-based DTI and DTA prediction methods are shown in Tables 3 and 4 below.

**Table 3:** **Summary of advantages and disadvantages of data representations**.

Data	Representations	Advantages	Disadvantages
Drug	String	Simple and convenient; Low storage space required;	Contain little information about the molecular structure; Difficult to handle due to its variable length, especially for CNN;
	Fingerprint	Variety of types to display a wide range of chemical properties; The capability of contain molecular structure information to some extent;	Can only be processed by sequence-level encoders; Diverse types and settings lead to the difficulty of unify;
	Graph	Rich in structural and chemical information; Favors structured studies in virtual screening, such as binding sites;	The selection of node and edge features; High storage space required;
Protein	String	Simple and convenient; Low storage space required;	Sequences contain limited information to explain the complex 3D structure of proteins; Sometimes too long which may contain tens of thousands of residues;
	Graph	Rich in structural and biological information; Favors structured studies in virtual screening, such as binding sites.	Contact maps do not accurately reflect 3D folding; Reliance on high-quality protein 3D structures; High storage space required.

**Table 4:** **Summary of advantages and disadvantages of deep learning-based virtual screening methods**.

Models	Advantages	Disadvantages
CNN	Effective extraction of local features of 1D structures of drugs and proteins; Weight sharing reduces the complexity of the network; Simple, stable and rapid training process;	Unable to capture the overall relationship of the data; The pooling layer may ignore the correlation between the local and the global; Limited by the fixed input size which padding and truncation operations can lead to information loss and noise;
RNN	The input length is variable, suitable for drug and protein sequences of arbitrary length; Good at handling time series information and able to consider the positional relationships of sequences;	Short-term memory problems and inability to handle longer inputs; Applies only to sequence data, ignoring other features of molecules and proteins; Interpretability deficiencies;
GNN	Capable of capturing spatial structural features of ligands and proteins; Convenient usage of abundant features; Relatively easy to combine with other deep learning techniques;	Prone to be oversmooth with the increasement of depth; Not suitable for training in batches which flexibility is limited; Not sufficient to make full use of edge features;
Transformer	The correlation between each element of the data can be calculated directly; Parallel calculation to make the best use of computational resources; Highly interpretable;	Weak local information acquisition compared to CNN and RNN; Difficulty in designing positional encoding to well characterize the positional information reliance on large-scale datasets;

### 5.2 RNN-based methods

RNN is a class of neural networks that operate recursively on sequence data. RNNs are well suited for processing sequence data such as languages. In virtual screening, RNNs have been successfully applied to protein sequences and Gene Ontology (GO).

Karimi et al. proposed DeepAffinity [110], which takes into account the possible dependencies between residues or atoms in the 3D structure, based on a seq2seq autoencoder architecture combining CNN and RNN, and incorporates an attention mechanism to gain information on the binding sites between drugs and proteins to improve the interpretability of the model. Gao et al. constructed embeddings of protein GO terms and amino acid sequences and projected sequential inputs of protein sequences into vector representations using Long Short-Term Memory (LSTM), and added an attention mechanism to provide biological interpretation of the model [57]. Wang et al. extracted the evolutionary features of proteins by using position specific scoring matrix (PSSM) and legendre moment, combining them with ligand molecular fingerprints to generate drug-target feature vectors, to finally constructed an LSTM model to complete the DTI prediction [111]. Mahdaddi et al. combined CNN with a BiLSTM based on attention mechanism, with the differential evolution Algorithm to optimize the hyperparameters [113]. The study proposed by Yuan et al. first introduced a pretrained language model to generate the input representation, then applied a two-layer BiLSTM to obtain the local and global dependencies of the feature vectors, and finally designed a multi-head attention layer to aggregate global information [114]. Tables 3 and 4 below list the computational methods for DTI and DTA prediction based on RNN.

### 5.3 GNN-based methods

Traditional CNN and RNN models represent drugs as strings of data format, but the structural information of molecules is lost as a result, which may reduce the predictive power of the model and the functional relevance of the learned potential space. GNN is adept at extracting the structural and spatial information of molecules and has been widely used in computational drug discovery, including interaction prediction, synthesis prediction, molecular design and quantitative structure prediction. Furthermore, with the rapid development of protein structure prediction, the usage of graph structure representation of proteins and the extraction of their spatial features based on GNN has become a major trend in virtual screening.

Ligands can be naturally represented as graph structures with atomic nodes and edges between the nodes, and GNN updates these node features while considering the neighbors of each node to extract the global structural features of the ligand. With the development of molecular graph representation techniques, an increasing number of DTI and DTA prediction models have recently applied GNN models, most of which use GNN as encoders for drugs. For instance, Tsubaki et al. first applied GNN in DTI prediction, using GNN and CNN to extract features of compounds and proteins, respectively [56]. The experimental results on two benchmark datasets, Human and *C. elegans* demonstrated the superior performance of this model. Feng et al. proposed PADME with two variants of ligand encoding in the context of the regression problem of DTA prediction [115]. PADME used ECFP encoding as well as graph representation, combined with protein feature vectors. On the other hand, Torng et al. introduced graph convolutional networks (GCN) into DTI prediction [116]. In their approach, residues in protein binding pockets correspond to nodes and the calculated feature vectors represent their physicochemical properties, while drug molecules are also converted into graph structures. A graph convolution layer is applied to each of these two graph structures and the resultant vectors of the two entities are connected and fed into a fully connected layer to understand the interaction between molecule and target to predict binding or nonbinding. Nguyen et al. proposed GraphDTA, using four GNN variants, including GCN, graph attention network (GAT), graph isomorphism network (GIN), and a combined GAT-GCN structure, to accomplish the drug affinity prediction task [117]. The performance obtained on the Davis and KIBA datasets indicated the potential of graph-based approaches in this field. Li et al. proposed a multi-objective neural network model named MONN [84], in which a graph warp module was specifically added to the traditional GCN model to learn the global features of the whole drug and the local features of individual atoms, while in the interaction prediction module, the method captured the noncovalent interactions between compound atoms and protein residues; thus, predicting the binding affinity. MGraphDTA proposed by Yang et al. introduced dense connectivity into GNNs and constructed an ultra-deep network structure to simultaneously capture the local and global structures of drugs [125].

For target proteins, various studies have been conducted to construct 2D or 3D graph structures and apply GNN. Jiang et al. first proposed a contact map-based protein graph method and applied it to DTA prediction [78]. The contact map is an output of the protein structure prediction method, usually in the form of a matrix, where each element indicates whether the corresponding residue pair is in contact or not. In addition, the team used various properties of amino acid residues and added PSSM as node features to finally obtain quite excellent prediction results. DrugVQA [79] and MINN-DTI [80] both used 2D distance maps to represent proteins, using CNN and transformer as protein encoders, respectively. Inspired by the above work, in 2022, both GEFA [81] and PSG-BAR [89] used contact maps to construct protein graphs and added pre-trained language model embeddings as the node features, which improved the interpretability of the models while obtaining better performance. STAMP-DPI [83], on the other hand, additionally added a contact map based on Hidden Markov Matrix (HMM), PSSM, and structural features, which were combined with the self-supervised protein representation method to obtain high-dimensional residue node features. Moreover, this model employed transformer decoder instead of the traditional fully connected layer as the final classifier to obtain a structure-aware multimodal DTI prediction model. Similarly, You et al. used 1D protein sequences and 2D contact maps as inputs, added pre-trained protein embedding representations, and proposed a cross-modal DTA prediction model [82]. The above studies were all based on contact or distance maps to construct residue graphs with protein amino acid residues as nodes. The study proposed by Liu et al. [123] constructed atom-level protein graphs with atoms as nodes and bonds as edges, used the graph transformer model, and designed an intermolecular attention mechanism to process protein, drug, and complex graphs separately, providing intermolecular information with more weight, which can better reflect the physical rules, and make the spatial relationship between drug and protein more robust, which significantly improves the performance. AttentionSiteDTI [88] proposed a different strategy, which calculated the protein binding pocket according to the algorithm of literature [140], and thus constructed an atom-level 3D protein binding pocket graph, improving the accuracy of protein map data and greatly reducing the computational resource consumption. The model also achieved accurate binding site prediction through an attention mechanism with advanced performance and high interpretability. From the above studies, it is likely that more virtual screening deep learning methods based on protein 3D structures and incorporating pre-trained embedding representations will emerge in the future and become one of the mainstream methods in this field.

All of the above models use different data formats to represent ligands and proteins separately, and subsequently make predictions of DTI or DTA from their representations, which are currently the dominant methods. However, a major drawback of such methods is the lack of network-level information, which means they ignore protein-protein interactions and drug-drug interactions. Another common class of methods is known as network-based methods [118]. Such methods calculate the similarity between drugs and targets based on the constructed network graphs, and have also received extensive attention in DTI prediction. Network-based DTI prediction methods utilize drug-protein interaction networks to predict interactions. The principle is that if two proteins are associated with similar structures and one can interact with a drug, the other can also interact with the drug. This approach usually involves constructing a network containing a drug and a protein and calculating the similarity scores of the drug and protein pairs. Networks containing drugs, proteins or both are usually constructed and GNN architectures are used to describe the complex interactions between different types of biological entities (i.e., compounds and proteins) [119]. Zhao et al. proposed GCN-DTI, a network-based DTI identification method using GCN [118]. GCN-DTI constructed a drug-protein pairs (DPP) network through a drug-drug interaction network, protein-protein interaction network, drug-protein pairs network. Subsequently, GCN-DTI transforms the DTI prediction problem into a node classification problem. Peng et al. used an end-to-end heterogeneous graph representation learning method and proposed EEG-DTI [119], which combined multiple biological networks to construct a heterogeneous network, while learning a low-dimensional feature representation based on the heterogeneous network and optimizing the model by end-to-end learning. Cheng et al. proposed GraphMS, where heterogeneous graph information is fused to obtain effective node information and substructure information based on interaction information, and an end-to-end autoencoder model is proposed to accomplish the DTI prediction task [121]. Li et al. proposed a multi-channel GCN and GAT-based model DTI-MGNN, which applied two independent GATs to learn different interactions between graph nodes of different strengths [120]. The authors then used a GCN-based architecture to learn the common information of both graphs and combined graph structure and semantic features to improve the representation learning capability of DPP. Sun et al. proposed an autoencoder-based approach to build a heterogeneous network to integrate drug, protein, and disease information [134]. The original drug features were mapped to a protein space through a multilayer encoder and then to a disease space through a decoder. The AEFS model proposed by this method preserved the consistency between chemical properties and functions of drugs, and the experimental results also showed that it possessed high robustness to unbalanced datasets. Table 3 lists the recent network-based deep learning methods for DTI prediction. The above studies jointly show that the structure of similar drug molecules, protein binding pocket residues, and protein-protein interaction networks can be effectively expressed using graph data structures.

### 5.4 Other methods

Attention mechanisms are gradually becoming more important in deep learning, including powerful NLP representation models such as transformer [129] and BERT [130], and are widely valued in bioinformatics. Zhao et al. first correlated attention mechanisms with the binding affinity of DTIs, using attention mechanisms to consider which subsequences in a protein are more significant for the drug and which subsequences in the drug are more significant for the protein, thus making the model more expressive [131]. Inspired by this, various virtual screening methods based on transformer have also been proposed to improve the application of the attention mechanism in DTI prediction. Wang et al. improved the work of Tsubaki et al. [56] and proposed the protein transformer model based on the attention mechanism as the protein encoder to capture the interactions between protein amino acid sequences [137]. TransformerCPI proposed by Chen et al. first transformed protein amino acid sequences into embeddings using the pre-trained Word2vec model, and then used GCN to learn the representation of each atom of the drug [132]. In TransformerCPI, the protein sequence is the input to the encoder, while the compound atomic sequence is the input to the decoder, and the output of the decoder is a vector of interaction features containing the same length as the atomic sequence. In contrast, MolTrans proposed by Huang et al. [59] extracted the substructure sequences of drugs and proteins separately, and each substructure was embedded in the potential feature vector, after which the substructure-embedded drug and protein sequences were input to the transformer encoder. The vectors obtained from the decoders were next fed into the classifier to obtain the interaction probabilities. In addition, Shin et al. introduced BERT to model the word and positional embedding of molecular sequences [133]. CoaDTI proposed by Huang et al. also leveraged the transformer as the encoder for protein sequences to obtain the global representation at the amino acid level [138]. Li et al. improved the transformer by introducing the interformer, where two interacting transformer decoders were used to extract feature vectors of targets and drugs [80]. TransDTI , on the other hand, combined various transformer-based protein and molecular pre-trained language models and obtained good performance in multiple classification and regression tests on multiple datasets [93]. FusionDTA also employed a pre-trained transformer instead of the original one-hot encoding of proteins, with distributed context vectors as the protein representation [114]. Therefore, use of the transformer-based pre-trained protein model instead of the original transformer can also attain good performance.

In addition to the attention-based deep learning models mentioned above, various types of generative models, such as variational autoencoder (VAE) and generative adversarial network (GAN), are also applied to DTI prediction. The autoencoder consists of two substructures, namely, an encoder and decoder, which can efficiently compress the input data and reconstruct the data into a compressed and simplified representation in an unsupervised setting. The approaches in the literature [122,^126^,134] are all autoencoder-based DTI prediction models. GAN is derived from game theory and contains two separate networks, i.e., the generator and discriminator, which act as targets against each other. GAN uses the discriminator network as a feature extraction network, which can also efficiently learn the potential representation of the input sequence. GANsDTA is a model that used GAN for DTA prediction [135]. All the above generative models can be used to extend the features of the input data. The various deep learning-based DTI and DTA prediction methods are summarized in Tables 5 and 6. The advantages and disadvantages of virtual screening methods using various deep learning models are summarized in Table 4.

**Table 5:** **Deep learning-based methods for DTI prediction**.

Models	Year	Ligand representation	Protein representation	Ligand encoder	Protein encoder	Description	Code availability
AtomNet [102]	2015	co-complex structure	co-complex structure	CNN	CNN	Using a CNN, composed of an input layer, i.e., the vectorized 3D grids, several 3D convolutional and fully-connected layers.	N/A
DL-CPI [103]	2016	fingerprint	sequence	DNN	DNN	A deep neural network model to extract features from chemical substructure and protein domain and predict DTI.	N/A
Ragoza et al. [104]	2017	co-complex structure	co-complex structure	CNN	CNN	A CNN based model to predict protein–ligand interaction with 3D depiction of co-complex structure.	https://github.com/gnina/models
MFDR [105]	2017	fingerprint	sequence	Autoencoder	Autoencoder	Using autoencoders as building blocks of deep network for reconstruct drug and protein features to low-dimensional new representations.	N/A
Tsubaki et al. [56]	2018	graph	sequence	GCN	CNN	Incorporating attention mechanisms into GNN of drugs to capture interaction sites.	https://github.com/masashitsubaki
Gao et al. [57]	2018	graph	sequence,Gene Ontology	GCN	LSTM	Combining RNN and GCN, adding an attention mechanism.	https://github.com/IBM/InterpretableDTIP
DeepConv-DTI [107]	2019	fingerprint	sequence	DNN	CNN	Predicting protein local residue patterns using CNN.	https://github.com/GIST-CSBL/DeepConv-DTI
Torng et al. [116]	2019	graph	graph	GCN	GCN	GNN-based learning of protein pockets and chemical structure graph representation.	N/A
DEEPScreen [109]	2020	SMILES	sequence	CNN	CNN	2D images of the drugs as input	https://github.com/cansyl/DEEPscreen
Wang et al. [111]	2020	SMILES	sequence	LSTM	LSTM	Based on LSTM and incorporating protein evolutionary features.	https://deepbiolab.coding.net/s/fbdb894d-2730–425b-bac6–18ba55396bab
DrugVQA [79]	2020	SMILES	graph	LSTM	CNN	Representation of proteins as distance maps, interpretable models based on quasi-visual question answering system.	https://github.com/prokia/drugVQA
MONN [84]	2020	graph	sequence	GCN	CNN	A multi-objective neural network model for predicting interactions and binding affinities.	https://github.com/lishuya17/MONN
GNN-PT [137]	2020	SMILES	sequence	GCN	CNN, Transformer	Constructing Protein Transformer to capture protein residue interactions.	https://github.com/JingtaoWang22/CPI_prediction
MolTrans [59]	2020	SMILES	sequence	Transformer	Transformer	Using the transformer encoder to extract the semantic relationships of data substructures.	https://github.com/kexinhuang12345/moltrans
TransformerCPI [132]	2020	SMILES	sequence	Transformer	CNN	Based on transformer, building novel datasets and introducing label inversion.	https://github.com/lifanchen-simm/transformerCPI
IGT [123]	2021	graph	graph	Graph Transformer	Graph Transformer	Constructing graphs of receptors, ligands and complexes, modelling intermolecular information using graph transformer.	N/A
HyperAttentionDTI [58]	2021	SMILES	sequence	CNN	CNN	Attention vectors are assigned to each atom and amino acid to simulate interactions.	https://github.com/zhaoqichang/HpyerAttentionDTI
TransDTI [93]	2022	SMILES	sequence	Transformer	Transformer	Simultaneous classification and regression prediction using multiple transformer-based pretrained language models.	https://github.com/TeamSundar/transDTI
BACPI [136]	2022	graph	sequence	GAT	CNN	Designing bidirectional attention mechanisms to integrate compound and protein representations.	https://github.com/CSUBioGroup/BACPI
MINN-DTI [80]	2022	graph	graph	CMPNN	Transformer	An interaction neural network is proposed by combining Interformer and CMPNN.	https://github.com/admislf/MINN-DTI
STAMP-DPI [83]	2022	graph	graph	GCN	GCN	Incorporating pretrained embedding representations in drug and protein graphs and constructing large-scale benchmark datasets.	https://github.com/biomed-AI/STAMP-DPI
AttentionSiteDTI [88]	2022	graph	graph	GAT	GAT	Construction of protein binding pocket graphs, with high interpretability using statement classification methods in NLP.	https://github.com/yazdanimehdi/AttentionSiteDTI
CoaDTI [138]	2022	graph	sequence	GraphSage	Transformer	Modelling of intra-modal and inter-modal interactions based on self-attention and co-attention mechanisms respectively.	https://github.com/Layne-Huang/CoaDTI
BridgeDPI [139]	2022	SMILES	sequence	CNN, GNN	CNN, GNN	Introduction of hyper-nodes to build protein/drug links.	https://github.com/DeepAAI/BridgeDPI
GCN-DTI [118]	2020	Drug-target network		GCN		Learning drug-target pair features using GCN.	https://github.com/zty2009/GCN-DNN
EEG-DTI [119]	2021	Drug-target network		GCN		End-to-end heterogeneous graph representation learning method.	https://github.com/MedicineBiology-AI/EEG-DTI
DTI-MGNN [120]	2021	Drug-target network		GCN, GAT		DTI prediction model based on multi-channel GCN and GAT.	https://github.com/catly/drug-target
GraphMS [121]	2021	Drug-target network		GCN		Adding mutual information to GNN.	N/A
GADTI [122]	2021	Drug-target network		GCN, autoencoder		Based on graph autoencoders, introducing GCN and matrix decomposition models	https://github.com/shulijiuba/GADTI
AEFS [134]	2021	Drug-target network		Autoencoder		Predicting DTI under spatial consistency constraints using an autoencoder architecture	https://github.com/JackieSun818/AEFS
GVDTI [126]	2022	Drug-target network		GCN, autoencoder		Separate encoding of graph representation, property representation, and property distribution for drug-target pairs.	https://github.com/pingxuan-hlju/GVDTI
KGE_NFM [127]	2022	Drug-target network		knowledge graph, recommendation system		Learning low-dimensional representations for various entities in the knowledge graph, and then integrates the multimodal information via neural factorization machine (NFM).	https://zenodo.org/record/5500305
HGAN [128]	2022	Drug-target network		heterogeneous graph attention network		Proposing the heterogeneous graph attention network to capture the complex structures and rich semantics in the biological heterogeneous graph for DTI prediction.	https://github.com/Zora-LM/HGAN-DTI

**Table 6:** **Deep learning-based methods for DTA prediction**.

Models	Year	Ligand representation	Protein representation	Ligand encoder	Protein encoder	Description	Code availability
DeepDTA [62]	2018	SMILES	sequence	CNN	CNN	Extraction of compound and protein sequence features using CNN.	https://github.com/hkmztrk/DeepDTA
WideDTA [106]	2018	SMILES	sequence, motif, domain	CNN	CNN	Incorporating chemical and biological sequence information features.	N/A
PADME [115]	2018	graph	sequence	GCN	DNN	Usage of GCN models eliminates the need for feature engineering.	https://github.com/simonfqy/PADME
DeepAffinity [108]	2019	SMILES	structural property sequence	RNN, CNN	RNN, CNN	Semi-supervised model combining RNN and CNN to predict DTA.	https://github.com/Shen-Lab/DeepAffinity
AttentionDTA [131]	2019	SMILES	sequence	CNN	CNN	Extracting drug and protein features using CNN and adding attention mechanisms.	N/A
MT-DTI [133]	2019	SMILES	sequence	Transformer	CNN	Proposing molecular transformer for better representation of SMILES.	https://mt-dti.deargendev.me/
Karimi et al. [110]	2020	graph	sequence	GCN	RNN	Combining GCN and RNN, adding an attention mechanism to enhance interpretability.	N/A
DGraphDTA [78]	2020	graph	graph	GCN	GCN	Constructing protein graphs based on contact maps and predicting structural features.	https://github.com/595693085/DGraphDTA
GraphDTA [117]	2020	graph	sequence	GCN, GAT, GIN	CNN	Applying multiple GNN models to graph representations of drugs.	https://github.com/thinng/GraphDTA
GANsDTA [135]	2020	SMILES	sequence	GAN	GAN	Semi-supervised GAN predicts affinity from protein and compound sequence data.	N/A
DeepDTAF [62]	2021	SMILES	sequence, structural property sequence	CNN	CNN	Adding protein binding pocket features to capture local and global contextual information.	https://github.com/KailiWang1/DeepDTAF
Mahdaddi et al. [113]	2021	SMILES	sequence	CNN, LSTM	CNN, LSTM	Fusing CNN and LSTM, identifying the optimal configuration using a differential evolutionary algorithm	N/A
SAG-DTA [124]	2021	graph	sequence	GCN	CNN	Proposing a self-attention graph pooling method to obtain representations of drugs	https://github.com/ShugangZhang/SAG-DTA
FusionDTA [114]	2021	SMILES	sequence	LSTM	LSTM, Transformer	Proposing a novel multiple attention mechanism instead of pooling, introducing knowledge distillation.	https://github.com/yuanweining/FusionDTA
MGraphDTA [125]	2022	graph	sequence	GCN	CNN	Constructing multi-scale ultra-deep GNN and visualizing the decision process.	https://github.com/guaguabujianle/MGraphDTA
GEFA [81]	2022	graph	sequence, graph	GCN	GCN	Combining sequence features and contact maps to construct protein graphs, fusing features using early fusion.	https://github.com/ngminhtri0394/GEFA
You et al. [82]	2022	graph	sequence, graph	GAT	RNN, GAT	Cross-modal models combining protein 1D sequences and 2D contact maps.	https://github.com/Shen-Lab/CPAC
PSG-BAR [89]	2022	graph	sequence, graph	GAT	GAT	Introducing an attention-based readout strategy to generate protein graph embedding representations.	https://github.com/diamondspark/PSG-BAR
MRBDTA [90]	2022	SMILES	sequence	Transformer	Transformer	Constructing the Trans block through improving the encoder of transformer and introducing skip connection at encoder level.	https://github.com/LiZhang30/MRBDTA

## 6 Comparison of performances of deep learning models

### 6.1 Evaluation metrics and strategies

To evaluate the performance of any deep learning method, it is common to split the data into training and test sets. The model is trained on the training set and evaluated by comparing the predicted labels with the given labels in the test set. In this section, we enumerate several common evaluation metrics and strategies for both classification and regression task in virtual screening. In addition, in Section 6.2 and 6.3, same metrics are used to calculate model performances.

For classification task, i.e., DTI task, the area under the ROC curve (AUC), and the area under the PR curve (AUPR) are used to evaluate the effectiveness of the model. For regression task, i.e., DTA task, the most common evaluation metrics are Concordance index (CI) and mean square error (MSE), which was utilized to calculate the difference between the predicted value of the model and the ground truth. The greater the CI the lower the MSE, and the better the prediction.

Cross-validation (CV) is often used to estimate prediction error and is typically performed using five or ten folds. The results are reported as mean performance (with standard deviation). Moreover, CV can also be used for hyperparameter tuning. It is worth noting that most of the existing methods in this domain perform a five-fold cross-validation (5-CV) on the dataset to ensure fair comparisons. Following this setup, the experimental comparisons in the following two sections were performed on the corresponding dataset under 5-CV setting.

### 6.2 Performance comparison on benchmark datasets

Table 7 summarizes the performance comparison of DTI prediction methods on benchmark datasets, using the AUC as the evaluation metric. Table 8 shows the performance of DTA prediction methods on the Davis and KIBA dataset as evaluated by the CI and MSE. Hyperparameters are based on the best results in the paper. The following data are taken from the references of each method and are taken as maximum values. It can be observed from the data that the performance of the models has gradually improved over time with the advancement of various data representation methods and deep learning models. The DTI prediction methods listed in Table 7 achieve high performance values on both datasets. Comparing the selected DTI prediction methods together, the better performing methods include MGraphDTA [125], AttentionSiteDTI [88], and BridgeDPI [139]. Among them, MGraphDTA and BridgeDPI both represented drug molecules as graphs and use the GNN model. AttentionSiteDTI constructed a 3D protein binding pocket graph to obtain better spatial features. When comparing the DTA prediction models in Table 8, the best performing methods include DGraphDTA [78], MGraphDTA [125], and FusionDTA [114]. Of these three high-performing methods, DGraphDTA used the contact map of the protein as its feature, while MGraphDTA and FusionDTA employed the sequence structure of the protein. From Tables 7 and 8, it can be observed that most of the current DTI and DTA prediction methods that have achieved superior performance use a custom GNN or transformer structure as the backbone network structure, which are currently the two most popular models in this field. Compared to the earlier methods based on CNNs and RNNs, there has been relatively more research using GNN models in the last two years, and they have been able to achieve better experimental performance. In addition, models based on transformer and attention mechanisms also have excellent performance, and the main reasons for this can be analyzed from two aspects: (1) Most CNN and RNN-based models use only the SMILES encoding of the compound as their features, and this 1D structure cannot represent the ligand effectively and may lose important structural information such as the adjacent nodes of the molecule in the network structure. (2) Most GNN-based models construct a graph structure for the ligand molecule, where the GNN can take into account the neighbor atomic information of each atom in the ligand molecule to represent the drug molecule more effectively. The attention mechanism in the transformer is also able to capture the overall structural information of the ligand molecule effectively and efficiently. In the future, how to combine the advantages of GNN and the transformer, which is also to capture both local and global information of drug molecules and proteins, and apply them to DTI and DTA prediction tasks, will remain a major research focus in virtual screening.

**Table 7:** **Comparison of AUC and AUPR metrics for DTI prediction methods**.

Methods	Year	Dataset			
		Human		*C.elegans*	
		AUC	AUPR	AUC	AUPR
DeepDTA [62]	2018	0.972	0.973	0.983	0.984
Tsubaki et al. [56]	2018	0.952	0.932	0.959	0.954
DeepConv-DTI [107]	2019	0.967	0.964	0.983	0.985
GNN-PT [137]	2020	0.978	0.973	0.986	0.982
DrugVQA [79]	2020	0.964	0.960	–	–
MolTrans [59]	2020	0.974	0.976	0.982	0.982
TransformerCPI [132]	2020	0.973	0.974	0.988	0.983
SAG-DTA [124]	2021	0.985	0.986	0.987	0.982
BACPI [136]	2022	0.978	0.964	0.989	0.981
MGraphDTA [125]	2022	0.983	0.980	0.991	0.986
AttentionSiteDTI [88]	2022	**0.991**	**0.990**	–	–
CoaDTI [138]	2022	0.982	0.975	0.985	0.980
BridgeDPI [139]	2022	0.990	0.984	**0.995**	**0.990**

^a^ ‘-’ indicates that the method was not tested on this dataset in the original papers.

^b^ Values in bold indicate the best results on the corresponding dataset.

**Table 8:** **Comparison of CI and MSE metrics for DTA prediction methods**.

Methods	Year	Datasets			
		Davis		KIBA	
		CI	MSE	CI	MSE
DeepDTA [62]	2018	0.878	0.261	0.863	0.194
WideDTA [106]	2018	0.886	0.262	0.875	0.179
DeepAffinity [108]	2019	0.900	0.253	0.842	0.188
AttentionDTA [131]	2019	0.893	0.216	0.882	0.155
MT-DTI [133]	2019	0.887	0.245	0.882	0.152
GraphDTA [117]	2020	0.893	0.229	0.891	0.139
GANsDTA [135]	2020	0.881	0.276	0.866	0.224
Karimi et al. [110]	2020	0.900	0.252	0.842	0.188
DGraphDTA [78]	2020	0.904	**0.202**	0.904	**0.126**
Mahdaddi et al. [113]	2021	0.893	0.235	0.876	0.179
SAG-DTA [124]	2021	0.903	0.209	0.893	0.131
FusionDTA [114]	2021	**0.913**	0.208	**0.906**	0.130
MGraphDTA [125]	2022	0.900	0.207	0.902	0.128
MRBDTA [90]	2023	0.901	0.216	0.892	0.146

### 6.3 Performance comparison on protein datasets of different sizes

#### 6.3.1 Construction of protein performance evaluation datasets of different sizes

**Fig. 3:**
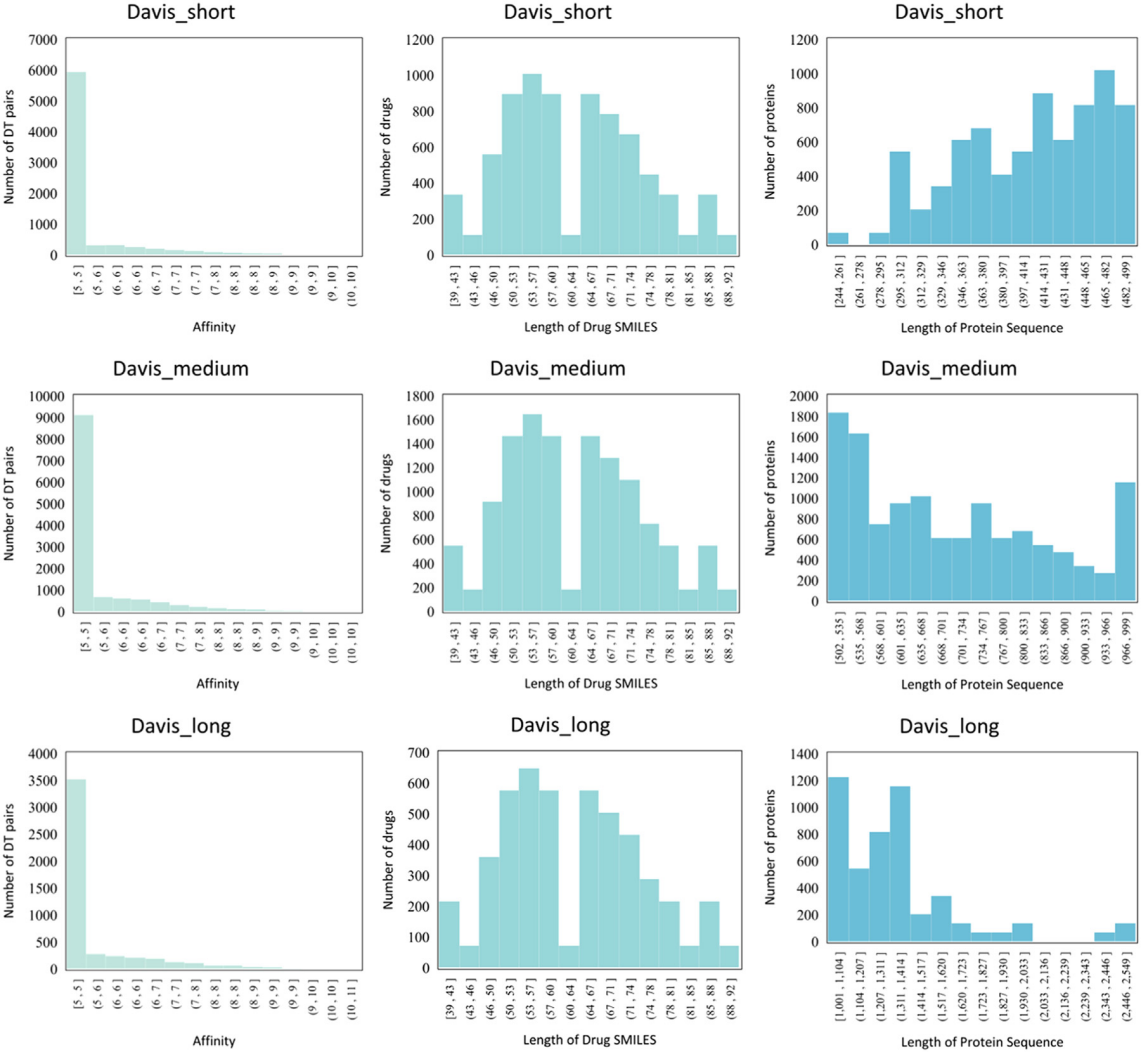
**The frequency histogram of binding affinity, length of drug SMILES, and length of protein sequence and in segmented Davis datasets**.

**Fig. 4:**
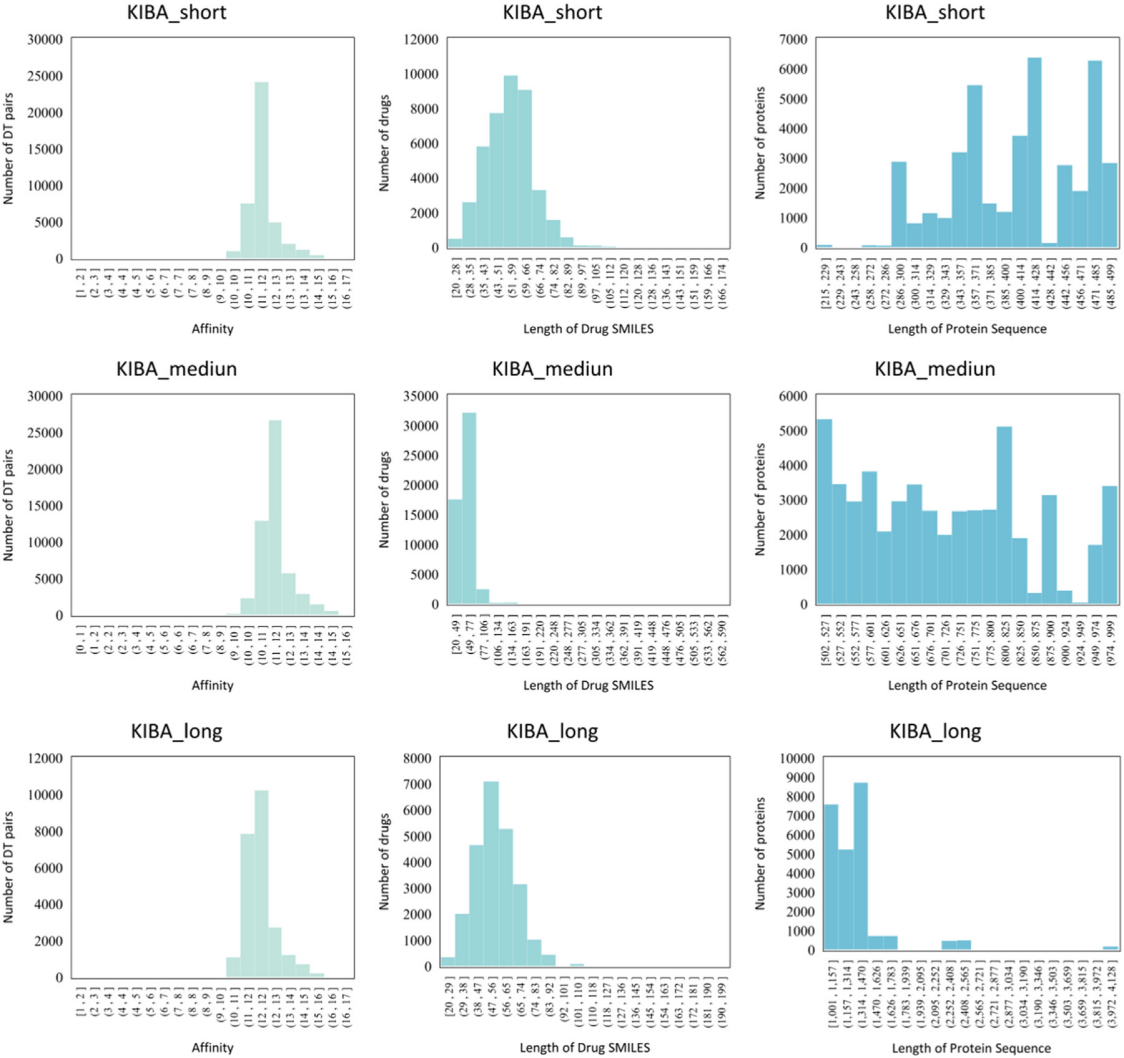
**The frequency histogram of binding affinity, length of drug SMILES, and length of protein sequence and in segmented KIBA datasets**.

**Fig. 5:**
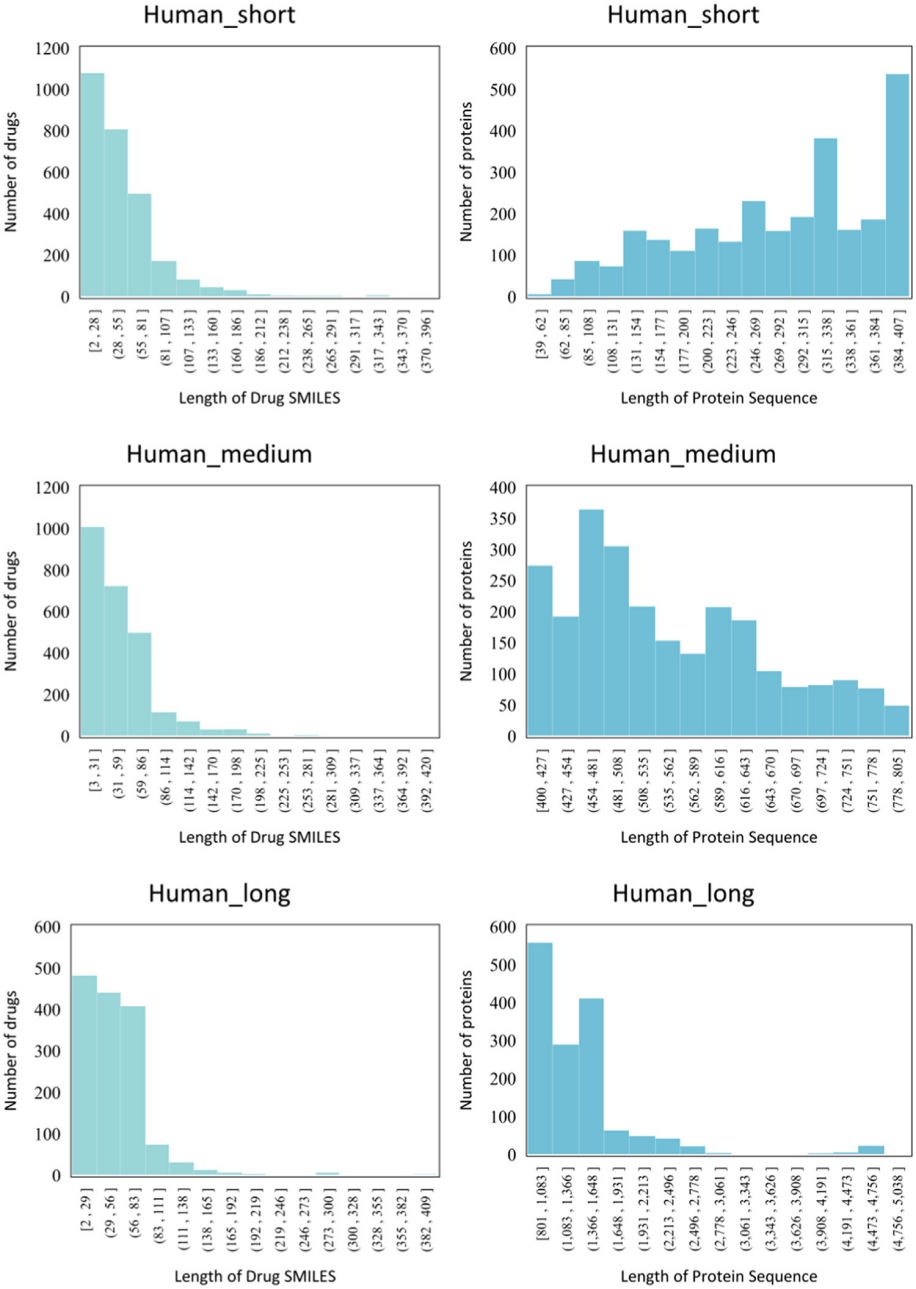
**The frequency histogram of length of drug SMILES, and length of protein sequence and in segmented Human datasets**.

**Fig. 6:**
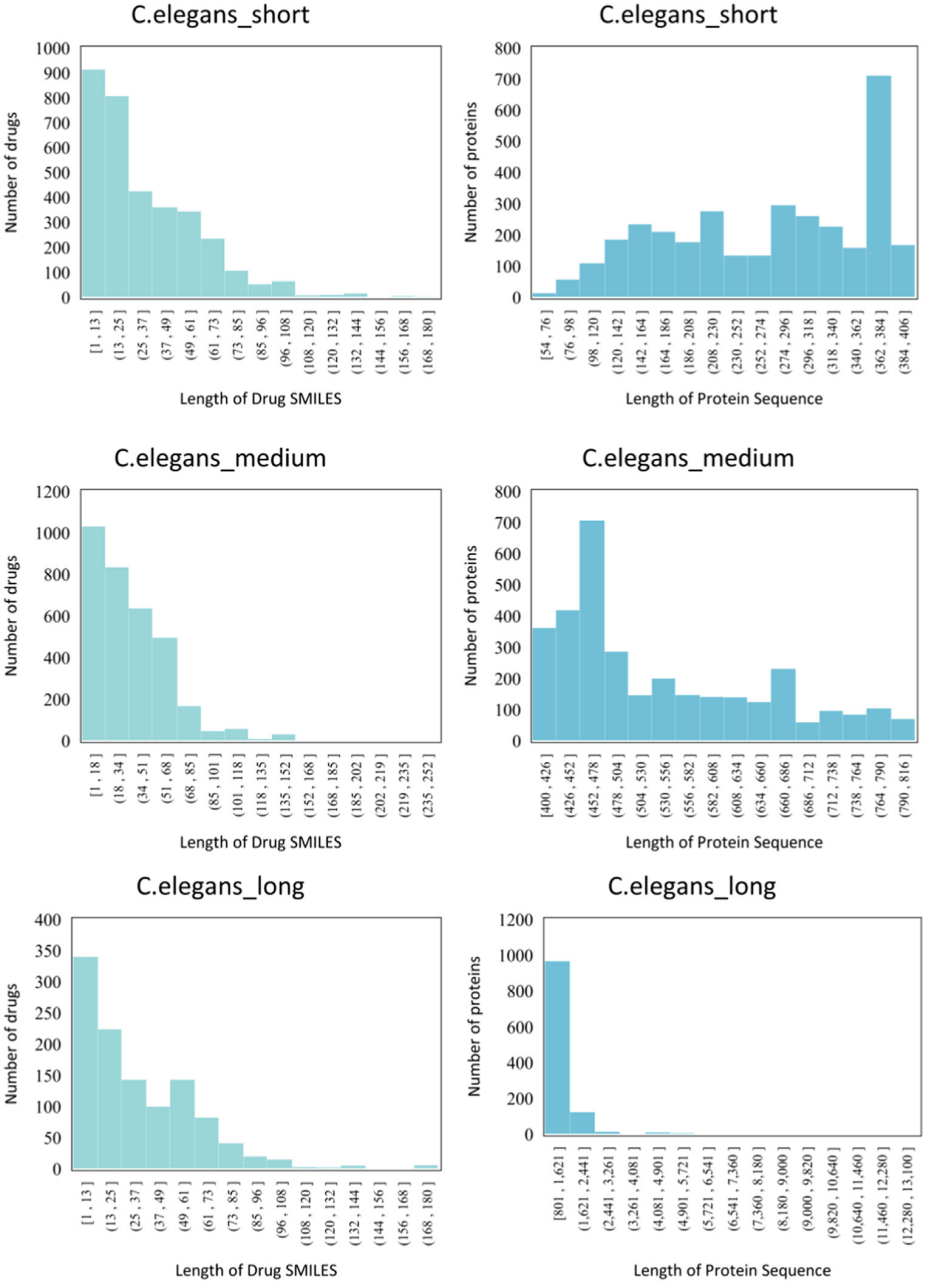
**The frequency histogram of length of drug SMILES, and length of protein sequence and in segmented *C.elegans* datasets**.

To investigate the performance of virtual screening methods based on different protein descriptors for proteins of different sequence lengths, we additionally partitioned the two benchmark datasets by protein length and selected several DTI and DTA models according to the protein descriptors used for performance testing on the new datasets. More specifically, we selected two benchmark datasets for the DTI and DTA tasks, including Human, *C. elegans*, Davis and KIBA, and segmented each of the four benchmark datasets by different protein lengths. For the DTI task dataset, we chose proteins of length 1 to 400 and filtered their interactions with ligands to construct the Human_short dataset and then selected proteins of length 401 to 800 and filtered their interactions with ligands to construct the Human_medium dataset. Finally, the Human_long dataset was constructed by selecting proteins with a length of more than 801 and screening their interactions with ligands. The *C.elegans* dataset was then segmented in the same way to obtain the *C.elegans*_short, *C.elegans*_medium and *C.elegans*_long datasets, respectively. As for the DTA task dataset, we adjusted the threshold value of the segmented data considering its protein length distribution. Specifically, we segmented the Davis and KIBA datasets according to the lengths of 1–500, 500–1000 and above 1000, and also obtained three new datasets representing short, medium and long protein lengths. Table 9 below shows the statistical information of the new datasets constructed by different lengths of proteins. Fig. 3, Fig. 4, Fig. 5–6 show the histogram of statistical information in the four new datasets respectively.

**Table 9:** **Comparison of statistical information for different length protein datasets**.

Task	Original dataset	Novel dataset	Drugs	Proteins	Interactions	Description
DTI	Human	Human_short	1,413	788	2,756	Constructed by selecting proteins of sequence length 1–400.
		Human_medium	1,405	826	2,502	Constructed by selecting proteins of sequence length 400–800.
		Human_long	804	387	1,470	Constructed by selecting proteins of sequence length 800 or more.
	*C.elegans*	*C.elegans*_short	984	802	3,347	Constructed by selecting proteins of sequence length 1–400.
		*C.elegans*_medium	1,115	738	3,314	Constructed by selecting proteins of sequence length 400–800.
		*C.elegans*_long	499	336	1,125	Constructed by selecting proteins of sequence length 800 or more.
DTA	Davis	Davis_short	68	111	7,616	Constructed by selecting proteins of sequence length 1–500.
		Davis_medium	68	183	12,444	Constructed by selecting proteins of sequence length 500–1000.
		Davis_long	68	71	4,896	Constructed by selecting proteins of sequence length 1000 or more.
	KIBA	KIBA_short	1,686	81	41,186	Constructed by selecting proteins of sequence length 1–500.
		KIBA_medium	2,063	104	52,357	Constructed by selecting proteins of sequence length 500–1000.
		KIBA_long	1,780	40	22,807	Constructed by selecting proteins of sequence length 1000 or more.

#### 6.3.2 Performance comparison

After constructing the completed novel dataset, we selected several DTI and DTA prediction methods and tested their performance on the new dataset. The selected DTI deep learning methods included: Tsubaki et al. [56], MGraphDTA [125], BACPI [136], BridgeDPI [139], MolTrans [59], TransformerCPI [132] and GNN-PT [137]. The selected DTA deep learning methods include DeepDTA [62], GraphDTA [117], MGraphDTA [125], DGraphDTA [78], FusionDTA [114] and MRBDTA [90]. We conducted the experiment according to the hyperparameters defined in the original paper. It should be noted that most of the above methods used the sequence information of the protein as an input feature without considering its 3D structure, whereas Tsubaki et al., MGraphDTA, BACPI, BridgeDPI, DeepDTA and GraphDTA all utilized CNN to process the protein sequence, while MolTrans, TransformerCPI and GNN-PT used transformer, and DGraphDTA is the only method that used GNN as the protein encoder, which are currently the dominant protein description methods in virtual screening methods. Furthermore, MolTrans was not tested on the Human and *C.elegans* datasets in the original article, while the other five methods chose the Human and *C.elegans* datasets as benchmark datasets. We additionally tested the MolTrans method on the Human and *C.elegans* datasets to better understand the performance changes on the novel dataset. Tables 10 and 11 below demonstrate the performance evaluation metrics obtained on the novel dataset for selected DTI and DTA prediction methods, respectively.

**Table 10:** **Comparison of AUC and AUPR metrics for selected DTI prediction methods on novel datasets**.

Methods	Protein encoders	Datasets											
		Human						*C.elegans*					
		Short		Medium		Long		Short		Medium		Long	
		AUC	AUPR	AUC	AUPR	AUC	AUPR	AUC	AUPR	AUC	AUPR	AUC	AUPR
Tsubaki et al. [56]	CNN	0.973	0.965	0.968	0.964	0.954	0.948	0.972	0.964	0.970	0.962	0.959	0.951
MGraphDTA [125]		**0.990**	**0.985**	0.968	0.966	**0.991**	**0.985**	**0.995**	**0.990**	0.989	0.981	0.971	0.970
BACPI [136]		0.976	0.974	0.969	0.962	0.971	0.961	0.989	0.985	0.982	0.973	0.965	0.962
BridgeDPI [139]		0.986	0.982	0.975	0.972	**0.991**	0.984	0.994	0.992	0.991	0.985	**0.982**	**0.976**
MolTrans [59]	Transformer	0.987	0.981	**0.980**	**0.977**	0.968	0.960	0.990	0.988	**0.992**	**0.987**	**0.979**	0.971
TransformerCPI [132]		0.969	0.962	0.968	0.966	0.970	0.967	0.981	0.976	0.983	0.979	0.973	0.971
GNN-PT [137]		0.979	0.977	0.979	0.973	0.972	0.964	0.984	0.980	0.980	0.973	0.977	**0.972**

**Table 11:** **Comparison of CI and MSE metrics for selected DTA prediction methods on novel datasets**.

Methods	Protein encoders	Datasets											
		Davis						KIBA					
		Short		Medium		Long		Short		Medium		Long	
		CI	MSE	CI	MSE	CI	MSE	CI	MSE	CI	MSE	CI	MSE
DeepDTA [62]	CNN	0.876	0.265	0.884	0.259	0.871	0.268	0.869	0.190	0.860	0.194	0.857	0.200
GraphDTA [117]		0.896	0.219	0.894	0.225	0.885	0.234	0.897	0.134	0.885	0.143	0.890	0.138
MGraphDTA [125]		0.898	0.208	0.902	0.206	0.896	0.212	0.900	0.126	0.903	0.130	0.902	0.129
DGraphDTA [78]	GNN	0.905	**0.202**	0.900	**0.200**	**0.905**	**0.203**	**0.902**	**0.123**	0.906	**0.124**	0.901	**0.126**
FusionDTA [114]	Transformer	**0.908**	0.204	**0.909**	0.205	**0.905**	0.206	0.901	0.128	**0.907**	0.127	**0.904**	0.129
MRBDTA [90]		0.902	0.212	0.900	0.218	0.898	0.218	0.890	0.138	0.893	0.141	0.892	0.142

Tables 10 and 11 indicate that most of the selected methods can achieve performance close to that of the original dataset for short protein lengths, while significant performance degradation is observed for medium and long protein lengths, especially for the long protein dataset. In terms of the chosen method, the performance degradation of the method by Tsubaki et al. is most pronounced on the long protein dataset, while MGraphDTA, BACPI, and BridgeDPI, which also use CNNs as protein descriptors, do not show significant degradation. In addition, the protein sequence length has a negligible impact on the performance of the three methods using transformer model, and DGraphDTA, which uses a contact graph representation of the protein and is based on GNN, also has a slight impact. The possible reason for the numerical decrease in performance on the long protein dataset is that all these methods use only the sequence of the protein as input and therefore do not obtain an accurate and effective representation and capture features when processing the data, as the information that can be obtained using only the 1D sequence of the protein is rather limited and important structural information may be lost during the processing to describe its complex folding and 3D structure of the protein. The impact is more pronounced when longer protein sequences are encountered compared to shorter and medium length proteins. Since both MGraphDTA and BACPI incorporate an attention mechanism, the performance degradation is smaller than that of Tsubaki et al. who also employed CNN as a protein encoder. Moreover, MolTrans, TransformerCPI and GNN-PT, which utilized transformer as the protein encoder, are less affected by protein length than CNN. This phenomenon is mainly due to the fact that CNN only captures local information of protein sequences, while the attention mechanism captures long-range interactions between amino acid residues to obtain overall information of the protein, which makes it more adaptable to different lengths of protein sequences and obviously has better robustness. Among the selected methods, DGraphDTA, which uses protein contact maps, also performs consistently on various protein length data sets, demonstrating that the graph-based approach can also cope with different lengths of protein data. It is clear that using sequence alone to represent proteins has obvious limitations, which ignores the complex structural and spatial information of proteins. However, most current DTI and DTA prediction methods use only the amino acid sequence of a protein, i.e., its primary structure, as the data representation, and only a few models consider the secondary and tertiary structure of a protein as the feature input. The application of protein secondary and tertiary structures to DTI and DTA prediction will also be a future research direction in virtual screening. With the significant breakthrough of AlphaFold2 in protein structure prediction, researchers can now obtain the 3D structure of proteins more conveniently. This research may lay an important foundation for the usage of protein 3D structures for DTI and DTA prediction in the future, and make it a major research topic in this field.

## 7 Conclusion and discussion

Over the past few years, substantial deep learning-based methods and applications have emerged in the field of virtual screening, particularly for drug-target interactions and affinity prediction. The development of virtual screening has been accelerated by the increased availability of bioactivity data, emergence of new data representation methods, and continuous updating and improvement of deep learning techniques. This paper details numerous recent deep learning-based virtual screening approaches, outlines the state-of-the-art and progress in the area, and lays the foundation for subsequent research and development. However, the rapid development of virtual screening is accompanied by several problems and challenges. We summarize the following six possible research directions for researchers' reference.

**Data representation.** The most widely used forms of ligand and protein representation are human-readable formats such as SMILES encodings and sequences. Nonetheless, these representations are often unable to carry critical information, such as neighborhood information in the 3D space of the molecule. Furthermore, as the data for training are not representative of the huge chemical space, the models lack generalization capabilities and simple memory patterns during training. The development of more effective methods of representing ligand molecules using graph structures, as well as the introduction of 3D structures of proteins, will be crucial research directions in the future. Recent strides in deep learning methodologies, particularly graph-based neural networks like GCN and voxel-based techniques such as 3D CNNs, have demonstrated notable success in capturing the intricate three-dimensional structures of molecules. However, challenges persist in handling diverse molecular structures and ensuring scalability for large datasets. Noteworthy advancements include the integration of molecular docking simulations with deep learning approaches like DeepDock, showcasing the potential to predict binding affinities more accurately. As 3D representation learning continues to evolve, it holds the promise of transforming ligand virtual screening, offering a nuanced understanding of molecular interactions and enhancing the efficiency of drug discovery processes within the DTI and DTA landscape.

**Imbalanced data impact.** The construction of decision boundaries for DTI predictions requires extremely complex computational methods, and the effects of negative interactions need to be excluded. Most of the benchmark datasets are balanced datasets with a close number of positive and negative samples. Many supervised learning methods simply treat all unlabeled drug-target pairs as negative samples, which may result in inaccurate predictions. In the real scenario, the majority of drugs are negative samples of a given target. Therefore, random over sampling is frequently used to increase the proportion of positive samples when training the DTI or DTA model, which may influence the generalization performance of the model in real-world prediction. Furthermore, the prediction performance of most models for experimental data is significantly lower than that for the DUD-E dataset, which is an extremely unbalanced dataset. Thus, the benchmark dataset may have a biased pattern for classifying active and inactive compounds, which is easily captured by deep learning models. There is high demand in the future for building high-quality unbiased benchmark datasets that consist of active and inactive drugs obtained from biological experiments. Another possible method to mitigate large labeled datasets is to pretrain the models on unlabeled data through self-supervised learning and then transfer the learned models to downstream tasks. Additionally, only a few studies on DTI and DTA prediction models have investigated the impact of unbalanced datasets on performance. To this end, some studies have been conducted to screen negative samples based on structural similarity, which also require further studies.

**Comparability of methods.** Research in any field needs to be compared to verify its effectiveness and to promote competition and development. Nevertheless, due to the large number of datasets in virtual screening and the distinction between classification and regression tasks, it is difficult to compare existing methods, even when using similar evaluation metrics. To achieve this, two key elements are required, namely open-source data and codes. Datasets and codes that are freely available and regularly updated will play an increasingly important role in virtual screening. It is also worthwhile investigating how to construct virtual screening datasets scientifically and rationally, and to open-source existing deep learning-based DTI and DTA prediction methods.

**Application of biomedical knowledge.** In the realm of virtual screening, diverse biomedical a priori knowledge, spanning genetics, genomics, transcriptomics, proteomics, and metabolomics, stands as a potent resource. However, the limited incorporation of such knowledge into deep learning models has left them lacking in biological significance and interpretability. A dearth of virtual screening methods employing mixed strategies further compounds this issue. A potential solution lies in amalgamating molecular structure with interaction networks, offering a holistic approach that considers chemical, biological, and medical knowledge concurrently. Additionally, the ‘black-box’ nature of deep neural networks, criticized for its interpretability deficit, hampers progress in the biomedical field. Models emphasizing biomedical interpretability not only enhance DTI predictions but also deepen our understanding of underlying mechanisms, paving the way for the discovery of novel drugs and targets. The fusion of biomedical and chemical property information with attention mechanisms emerges as a promising avenue for comprehensive advancements. Future research should delve into automating the correlation between machine learning results and existing biomedical knowledge or wet laboratory experimental outcomes. To augment the biological significance of models, incorporating additional biomedical data such as gene expression and protein interactions is essential. Exploring mixed strategies that integrate chemical and biomedical data offers a more holistic approach to virtual screening. Furthermore, enhancing model interpretability through interpretable deep learning or rule-based methods can significantly contribute to the integration of machine learning into biomedical research, providing more nuanced and understandable insights for drug discovery and disease comprehension.

**Generalization of models.** A model with high generalization can provide equally reliable results for unknown test data. Although some deep learning models perform well in this domain, there still seem to be challenges in exploring the entire chemical and biological space. Some models have shown less successful results when evaluated on external datasets or encountered unseen data, indicating that since the training data does not represent the vast chemical and biological space, the model is not learning and exploring it but rather memorizing patterns from the training data, which contribute to the generalization issue. Only a few researchers have focused on datasets with different distributions of training and test data. Deep transfer learning, which transfers knowledge from one model to another and using domain adaptation techniques to map the source and target data into new representations with the same distribution, may be an effective way to address this issue. Also, it would be a potential development direction for other methods to improve the generalization ability of models.

**Large language model integration.** Large language models (LLMs), exemplified by ChatGPT, play a pivotal role in advancing DTI research, ushering in transformative changes in the computational landscape of drug discovery. These models are instrumental in predicting and comprehending complex DTIs, providing valuable insights into binding affinities and interaction mechanisms. By streamlining the early stages of target identification and validation, LLMs significantly contribute to expediting the drug discovery pipeline. Their prowess lies in capturing semantic understanding and contextual information, offering nuanced insights into the intricate relationships between drugs and targets. The rise of domain-specific LLMs, as demonstrated by DrugChat, further refines their application, addressing challenges associated with generalization. Looking ahead, the integration of LLMs in DTI research is poised for continual evolution, focusing on enhancing predictive accuracy, interpretability, and fostering collaborative efforts with experimental validation for robust and reliable insights into drug-target interaction studies.

## Declaration of competing interest

The authors declare that they have no conflicts of interest in this work.
